# Single-Molecule SERS Detection of Phosphorylation in Serine and Tyrosine Using Deep Learning-Assisted Plasmonic Nanopore

**DOI:** 10.1021/acs.jpclett.5c01753

**Published:** 2025-08-08

**Authors:** Mulusew W. Yaltaye, Yingqi Zhao, Kuo Zhan, Eva Bozo, Pei-Lin Xin, Vahid Farrahi, Francesco De Angelis, Jian-An Huang

**Affiliations:** † Research Unit of Health Sciences and Technology, Faculty of Medicine, 6370 University of Oulu, Aapistie 5 A, 90220 Oulu, Finland; ‡ Research Unit of Disease Networks, Faculty of Biochemistry and Molecular Medicine, 6370 University of Oulu, Aapistie 5 A, 90220 Oulu, Finland; § Biocenter Oulu, 6370 University of Oulu, Aapistie 5 A, 90220 Oulu, Finland; ∥ Institute for Sport and Sport Science, 14311 TU Dortmund University, Dortmund 44227, Germany; ⊥ 121451 Istituto Italiano di Tecnologia, Via Morego 30, 16163 Genoa, Italy

## Abstract

Single-molecule detection of post-translational modifications (PTMs) such as phosphorylation plays a crucial role in early diagnosis of diseases and therapeutics development. Although single-molecule surface-enhanced Raman spectroscopy (SM-SERS) detection of PTMs has been demonstrated, the data analysis and detection accurracies were hindered by interference from citrate signals and lack of reference databases. Previous reports required complete coverage of the nanoparticle surface by analyte molecules to replace citrates, hampering the detection limit. Here, we developed a high-accuracy SM-SERS approach by combining a plasmonic particle-in-pore sensor to collect SM-SERS spectra of phosphorylation at Serine and Tyrosine, k-means-based clustering for citrate signal removal, and a one-dimensional convolutional neural network (1D-CNN) for phosphorylation identification. Significantly, we collected SM-SERS data with submonolayer analyte coverage of the particle surface and discriminated the phosphorylation in Serine and Tyrosine with over 95% and 97% accuracy, respectively. Finally, the 1D-CNN features were interpreted by a one-dimensional gradient feature weight and SM-SERS peak occurrence frequencies.

## Graphical abstract

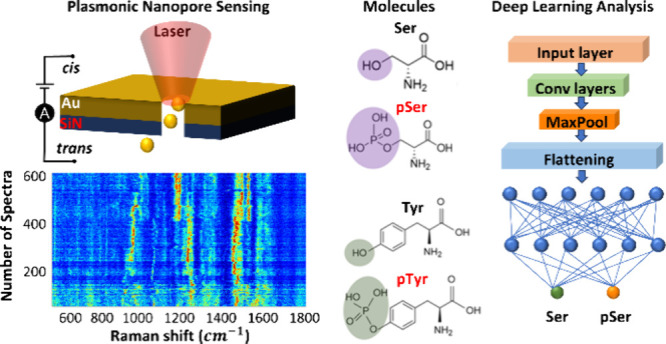

Post-translational modifications (PTMs) play essential roles in protein signaling, function, localization, and other important biological processes.^1^ Most PTMs attach small chemical groups, such as phosphoryl (153.32 Da), to amino acids of proteins, which can ultimately cause serious health consequences.^2,3^ Phosphorylation is a ubiquitous PTM in which a phosphate group is covalently attached to a specific amino acid residue, which has a significant impact on vibrational modes.^4^ Phosphorylation events of serine (Ser) and tyrosine (Tyr) residues modulate critical signaling pathways in eukaryotic cells, which can provide insight into the mechanisms underlying cancer development and serve as potential therapeutic targets for developing new drugs or therapies for prostate cancers.^5,6^ Phosphorylation at serine residues has been implicated in several physiological and pathological processes, including insulin signaling, tumor progression, inhibition of pathological calcification, and modulation of inflammatory and oncogenic pathways in metabolic disorders such as diabetes, chronic kidney disease, and cancer.^7^ On the other hand, phosphorylation at tyrosine plays a critical role in the pathogenesis of Parkinson’s disease (PD).^8,9^

Mass spectrometry is currently the main technology for phosphorylation analysis, but its sensitivity is limited by the requirement of 10^6^–10^8^ copies of molecules.^10^ On the other hand, fluorescent sensors offer single-molecule sensitivity but often rely on antibodies or fluorescent tags, which can limit their specificity and applicability. Surface-Enhanced Raman Scattering (SERS), which is highly sensitive to molecular vibrations, has emerged as a powerful analytical tool for detecting protein PTMs at the single-molecule level.^11–13^ However, conventional SERS substrates based on nanoparticles suffer from the strong signals of aromatic amino acids, which can overwhelm the signals of PTMs at non-aromatic amino acids.^14,15^ In contrast, the plasmonic particle-in-pore sensor (Figure 1a) trapped a gold nanoparticle in a gold nanopore to generate a single, ultrasmall gap-mode plasmonic hot spot able to detect both aromatic and non-aromatic amino acids in single peptides.^16,17^ The high spatial and spectral resolution of the particle-in-pore sensor would be promising to detect small PTMs such as proline hydroxylatioin in amino acids, peptides, and even at the protein level.^18^

**Figure 1:**
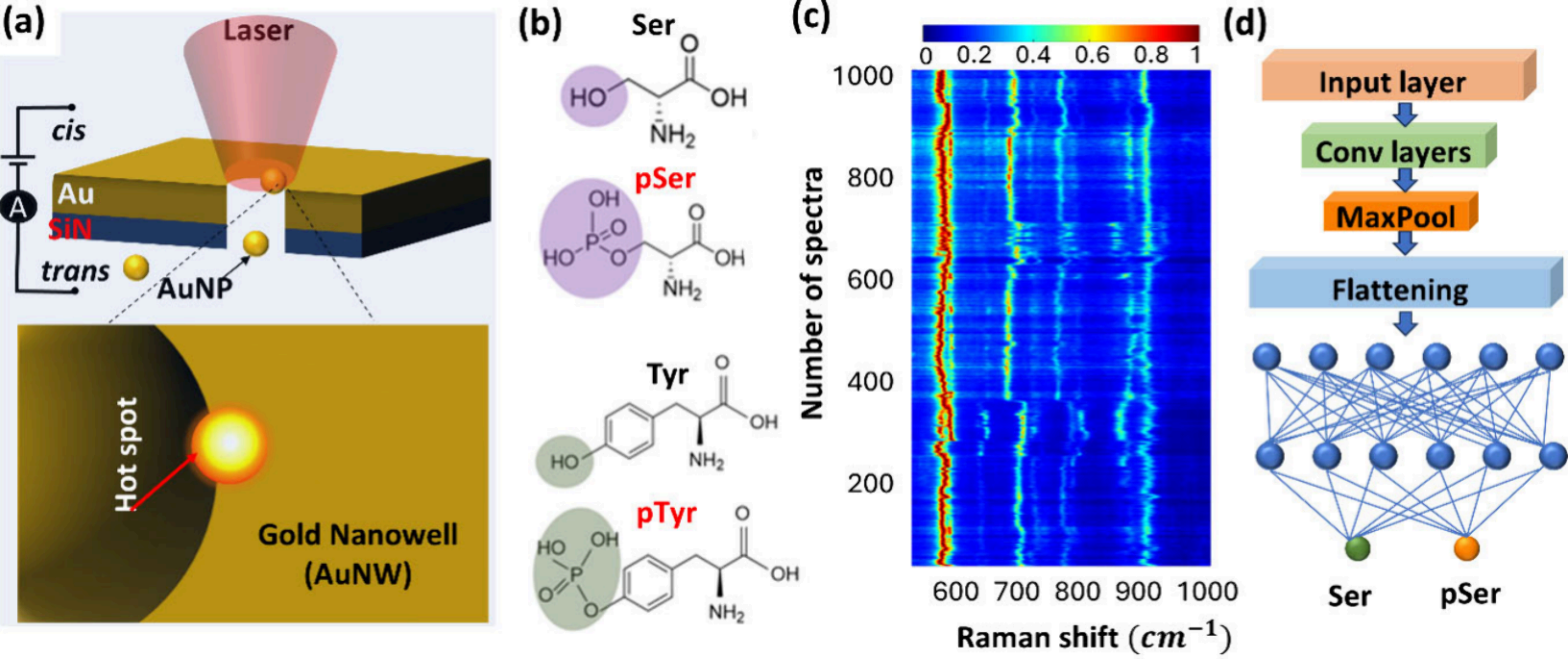
Schematic of the deep learning-assisted SERS method for single-PTM detection. (a) Schematic of the plasmonic particle-in-pore sensor with a hot spot that excites the molecule or part of the molecule. (b) Molecular structures of Ser, pSer, Tyr, and pTyr. (c) Heatmap plot of the SM-SERS spectra time series. (d) 1D-CNN deep learning model for phosphorylation identification.

Nevertheless, single-molecule SERS (SM-SERS) analysis of phosphorylation is challenging by the traditional analysis method that identifies the phosphorylation by the shift and intensity change of the SERS peaks based on a SERS database. The method relying on the databased from multimolecule data becomes unreliable to analyze the SM-SERS spectra with continuous peak shifts and intensity fluctuations commonly referred to as ″blinking″, which arise from the Brownian motion and dynamic conformational changes of the molecule within the plasmonic hotspot.^16,19,20^ In addition, the citrate, commonly used as a stabilizing surfactant in the nanoparticle system, seldom generates background signals in the multimolecule SERS system of nanoparticles, because they were completely replaced by the analyte molecules on the nanoparticle surface. In strong contrast, the citrate signals significantly interfered with SM-SERS signals from the SM-SERS sensor where analyte molecules were adsorbed on the nanoparticle surface in a submonolayer manner, because the citrates would compete with target analytes for adsorption sites in the hot spot.^18,21^ Other research groups used oxygen plasma to remove the citrates from the enhancing surface of the SM-SERS sensor,^22^ which limits the applications. Finally, the lack of single-molecule SERS databases of pure pSer and pTyr places another barrier.

In this work, we developed a high-accuracy SM-SERS approach by combining a plasmonic particle-in-pore sensor (Figure 1a) to collect SM-SERS spectra of phosphorylation at Serine and Tyrosine (Figure 1b), k-mean clustering for citrate signal removal, and a one-dimensional convolutional neural network (1D-CNN) for phosphorylation identification. While the particle-in-pore SERS sensor provides single hot spot with high localization to detect SM-SERS signals with ultralow analyte coverage of 1.23% particle surface (Figure 1c), citrate-interfered spectra were effectively precluded by k-means clustering before training and post-evaluation of the 1D-CNN model.^23^ The customized 1D-CNN model consists of hierarchical convolutional layers and pooling operations (Figure 1d), which enables extraction of subtle spectra differences of phosphorylation from the blinking single-molecule SERS data (Figure 1c).^24,25^ Taking the advantage of large amount of single-molecule SERS data acquired from the particle-in-pore sensor, the synergy of the particle-in-pore sensor with the 1D-CNN model would allow automated information extraction from complex spectra for single-molecule PTM analysis,^26–28^ which overcame the above-mentioned drawbacks of traditional SERS analysis. Notably, we have achieved an overall accuracy of over 95% for the identification of Ser from pSer, and 97% for the identification of Tyr from pTyr. This work demonstrated the high-accuracy SERS detection of pSer and pTyr at the single molecule level. This is a significant step toward single-molecule PTM analysis, which has vast applications in personalized medicine, drug discovery, and therapeutic intervention monitoring.

The plasmonic particle-in-pore sensor with SM-SERS sensitivity was fabricated to collect the SM-SERS spectra of PTMs according to the protocol in our previous papers.^16,17^ The gold nanopores of 200 nm diameter were fabricated on a silicon nitride (SiN) membrane by Focused ion Beam milling (see fabrication details in Supporting Information). After adsorbing 1/80 monolayer, i.e., 1.23% of the particle surface area, of analyte molecules on the gold nanoparticle (AuNP) of 50 nm diameter (details in the Supporting Information Table S1), the nanoparticle was trapped in the nanopore for minutes under 785 nm laser illumination by the ThermoFisher DXR2xi Raman microscope with 15 mW laser power, slit width of 50 μm, and 0.1 s exposure time. Consequently, a single plasmonic hot spot with a strong field enhancement was generated on the nanoparticle to excite the molecule and emit SM-SERS signals.

The SM-SERS spectra of Ser, Tyr, and their phosphorylation products, phosphorylated serine (pSer) and phosphorylated tyrosine (pTyr), were collected by the particle-in-pore sensor. The SM-SERS measurements in our particle-in-pore platform are quite reproducible, as we collected more than 20,000 SM-SERS spectra for each molecule (Citrate, Ser, Tyr, pSer, pTyr). We used 10 nanopores for each type of molecule. For an independent measurement, a new particle would be trapped in the nanopore to produce the SM-SERS spectra time series. We had considered 13 independent measurements for citrate, 11 for tyrosine, 23 for phosphorylated tyrosine, 13 for Serine, and 15 measurements for phosphorylated serine. Each independent measurement contains 2000 spectra, while some yield a better signal than others, depending on the molecular structures. Typical preprocessed SM-SERS spectra time series are shown in Figure 1c processed by the SERS signal processing pipeline in Supporting Information Figure S1.

Unlike multimolecule SERS spectra where spectral features are averaged over many molecules, SM-SERS spectra exhibit significant temporal fluctuations in peak position, intensity, and bandwidth. This variablility is due to the probabilistic nature of molecular adsorption within the hot spots.^29^ Additionally, peak shifts can occur due to charge transfer interactions between the target molecule and the gold surface in different orientations.^30^ Despite these fluctuations, SM-SERS retains high chemical specificity, capable of resolving subtle differences in molecular structure, isotopic composition, and even intermolecular interactions.^31^ Instead of using peak intensity, we apply a histogram of SM-SERS peak occurrence frequency, for example, in Figure 2a,b (pSer) and c,d (pTyr), to visualize and emphasize the narrow, continuously occurring SM-SERS peaks. They characterized the most probable molecular conformation of the analyte molecules and citrates within the plasmonic hot spot.^32,33^ Similarly, the SM-SERS spectra of Tyr and Ser and their peak occurrence frequencies can be found in Supporting Information Figure S2.

**Figure 2:**
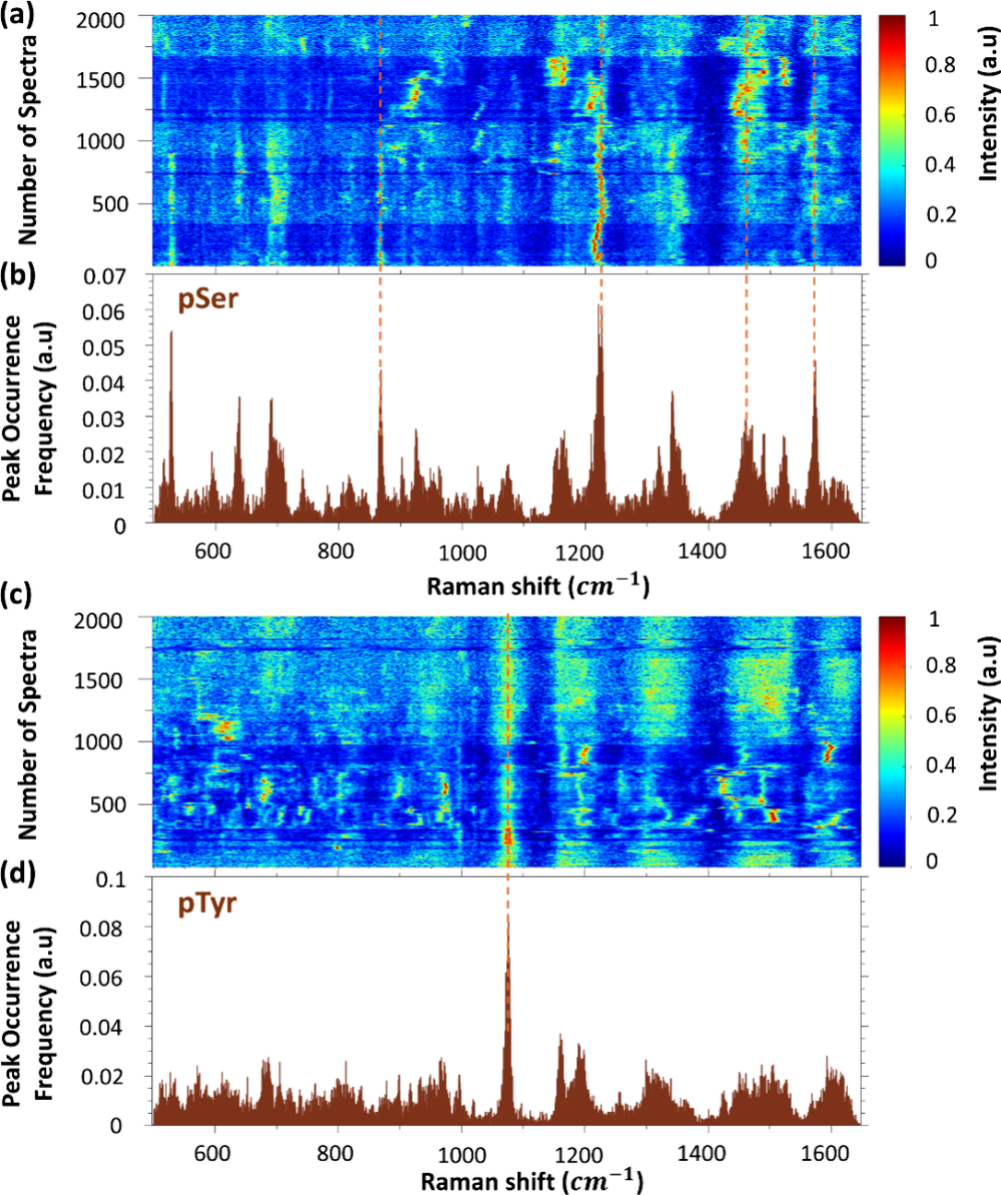
Examples of SM-SERS spectra and histograms of SM-SERS peak occurrence freqauency. (a) Fluctuating SM-SERS spectra of pSer, with the red line indicating the most frequently occurring Raman shift. (b) SM-SERS peak occurrence frequency of pSer. (c) Fluctuating SM-SERS spectra of pTyr. (d) SM-SERS peak occurrence frequency of pTyr.

In our previous paper, we identified hydroxylation at the single molecule level,^18^ whereas this work focuses on the phosphorylation of serine and tyrosine. The citrate interference is a bottleneck for single molecule data analysis. Previously, we experimentally mitigated this issue by substituting citrate with an analyte monolayer. However, this approach has two key limitations. First, it does not completely eliminate citrates, leading to residual interference, as citrate could still access the hot spot. Second, the requirement of a substantial number of analyte molecules to form a complete monolayer on the nanoparticle limits further improvements in the detection sensitivity.

To address these limitations, we engineered the platform such that the analyte molecule occupied only 1.23% of the particle surface. The remaining surface was covered by citrates, which generated SM-SERS noise when it was excited by the hot spot. To preclude citrate influence, we implemented a k-means-based clustering algorithm to exclude citrate-contaminated spectra from our SM-SERS data sets. The k-means-based clustering has three stages: (1) clustering with the k-means algorithm; (2) identification of the contaminated cluster (i.e., the cluster containing most of pure-citrate spectra); and (3) identification of citrate-affected spectra from the target molecule by iteratively searching within the contaminated cluster. First, SM-SERS spectra of pure citrates are collected using the bare nanoparticles (i.e., only citrate surfactants on the particle surface) in the particle-in-pore platform. Second, by using 2051 spectra of pure-citrate as a reference, we implemented k-means algorithms in MATLAB to cluster SERS spectra. k-Means clustering minimizes within-cluster variance by iteratively assigning spectra to their nearest cluster centroids based on spectral similarity metrics.^34^ We considered three clusters based on our prior knowledge and optimal numbers of cluster using the elbow method in Figure S3 in the Supporting Information. Following k-means clustering in Figure 3b and e, we identified the cluster predominantly composed of citrate spectra (contaminated cluster) using the “mode” function in MATLAB. This identified cluster was subsequently established as a reference database to effectively filter out any citrate-affected analyte spectra. The contaminated cluster primarily contains spectra of citrate, along with citrate-affected spectra from the analytes. We identified and removed any citrate-affected target molecule spectra that had been assigned to this contaminated cluster using “find” function in MATLAB.

**Figure 3:**
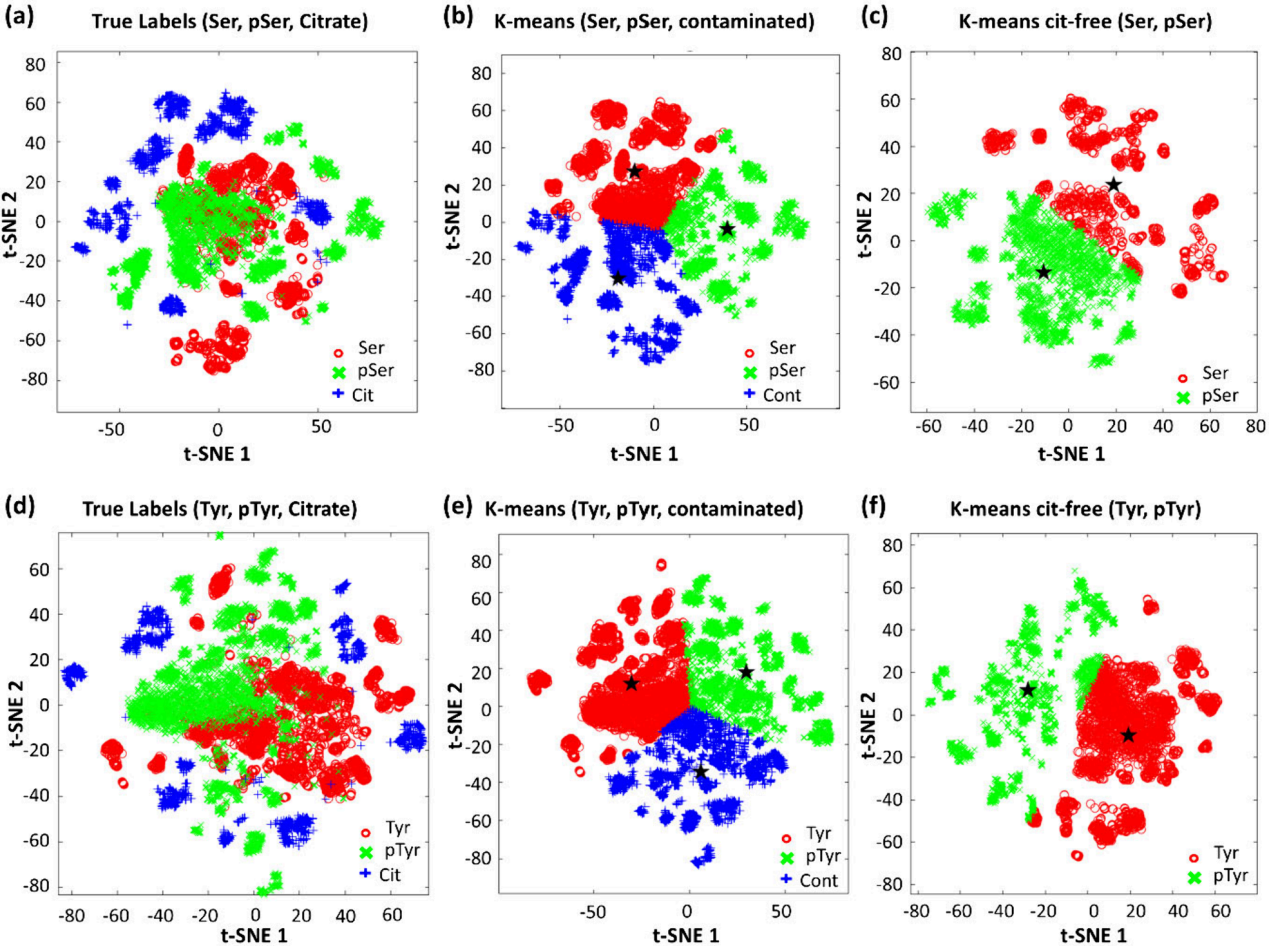
Workflow of k-means-based clustering. The black star (★) indicates the centroid in each cluseter. (a) t-SNE visualization of Ser, pSer, and citrate (Cit) in two-dimensional space; (b) t-SNE visualization of k-means clustered spectra of Ser, pSer, and citrate-contaminated (Cont) data; (c) t-SNE visualization of citrate-free, k-means clustered Ser and pSer spectra; (d) t-SNE visualization of Tyr, pTyr, and citrate (Cit) in two-dimensional space; (e) t-SNE visualization of k-means clustered Tyr, pTyr, and citrate-contaminated (Cont) spectra; and (f) t-SNE visualization of k-means clustered, citrate-free spectra of Tyr and pTyr.

To visualize the different clusters distinctly, we first implemented principal component analysis (PCA) to reduce the dimensionality of the original spectra. Each spectrum initially contained 1048 features (Raman shift intensity values), which we reduced to the 30 most significant principal components. We then applied t-distributed stochastic neighbor embedding (t-SNE), a robust non-linear dimensionality reduction technique, to visualize the spectral clustering outcomes as distinct groups in a two-dimensional space. (Detailed implementation available in the Supporting Information.)

As a result, some spectra from Ser, pSer, Tyr, and pTyr are reassigned as contaminated, meaning that these spectra are highly correlated to the citrate or strongly affected by citrate. We have presented the original, clustered, and citrate-free data sets of Ser and pSer in Figure 3a-c. Similarly, the t-SNE visualization of Tyr and pTyr before and after citrate removal using a k-means-based clustering algorithm is shown in Figure 3d-f. We quantified the relative number of spectra in each cluster in Figure S4 and the profile of spectra precluded by the k-means-based algorithm in Figure S5 in the Supporting Information. PCA, a linear technique, focuses on the global structure of the data. In contrast, t-SNE maintains local data relationships by constructing a probability distribution that reflects pairwise spectral similarities in the high-dimensional space.^35^ Subsequently, it optimizes a low-dimensional embedding that preserves these intricate spectral relationships.^36^ Thus, the integration of k-means clustering with an iterative search of individual spectra within the citrate database effectively mitigates citrate-induced interference, ensuring the selection of SERS spectra for precise molecular characterization.^37^

Using the k-means-based citrate removal technique, we developed a citrate-free subset data set: subsequently we developed and validated a customized 1D-CNN model. Our 1D-CNN model consists of an input, three convolutional blocks, a flattening layer, two fully connected blocks (dense blocks), and an output layer. Each convolutional block consists of two convolutional layers, two batch normalization (BN) layers, the max-pooling layer, and dropout layers. We started a convolutional layer with 16 filters and proceeded to a convolutional layer having 64 filters by doubling each time. Batch normalization accelerates convergence, while max-pooling reduces spatial dimensions, and dropout prevents overfitting. The flattening layer is used to transform multiple feature maps produced by the convolutional layers into a 1D vector. The rectifier linear unit (ReLU) activation function introduces non-linearity to the model and mitigates the vanishing gradient and l2 kernel regularization is used. The fully connected (Dense layer) basically makes the final decision after the convolutional and pooling layers extract features, which should learn the global patterns in their input feature spaces to classify them. Finally, the Sigmoid activation function in the output layer is used to convert raw score outputs into a probability distribution over the binary classes (detailed in Figure S6 in Supporting Information). CNN has been applied for spectroscopic data analysis due to their excellent performance in feature extraction and identification problems.^38–40^ We implemented a 1D-CNN in python version 3.11.5 with the TensorFlow framework (details in Table S3 of the Supporting Information) to distinguish Ser from pSer and Tyr from pTyr using 1D-CNN based on their SM-SERS spectra.

A total of 2572 spectra of Ser, 3803 spectra of pSer, 4231 spectra of Tyr, and 5108 spectra of pTyr were extracted and divided into training, validation, and post-evaluation sets. For each identification task, two data sets were prepared: the original data set (citrate-affected data set) and a citrate-free data set. The citrate-free data set was obtained by removing citrate-contaminated spectra using the k-means clustering algorithm, resulting in 1605 spectra of Ser, 2281 spectra of pSer, 2484 spectra of Tyr, and 3125 spectra of pTyr. Each data set was randomly split into training and validation sets in a 70:30 ratio. Model performance was subsequently evaluated using an unseen data set, referred to as the post-evaluation set, as summarized in Table S2 of the Supporting Information.

The classification accuracies of the 1D-CNN model on the validation and post-evaluation sets evaluated by the confusion matrix are shown in Figure 4a for Ser vs pSer identification based on citrate-affected data set, and in Figure 4b for the citrate-free data set. We achieved post-evaluation accuracies of over 81% for the citrate-affected data set and over 93% for the citrate-free data set, indicating that the use of the k-means clustering algorithm to remove citrate-contaminated spectra significantly improves classification performance. Figure 4c shows their Receiver Operating Characteristic (ROC) curves, demonstrating high sensitivity and specificity. We have presented the area under the curve (AUC), precision, and recall values from both training and post-evaluation in Supporting Information Table S4. The model’s training performance on the validation of citrate-free and citrate-interfered data sets during the training were in the Supporting Information Figure S7. However, the accuracies and ROC curves for Tyr versus pTyr in Figure 4d-f before and after citrate signal removal do not show such a significant increase in accuracy. It could be due to the aromatic molecular structure of Tyr and pTyr against the non-aromatic molecular structure of Ser and pSer, because the former can generate much stronger SM-SERS signals than the citrate ones.

**Figure 4:**
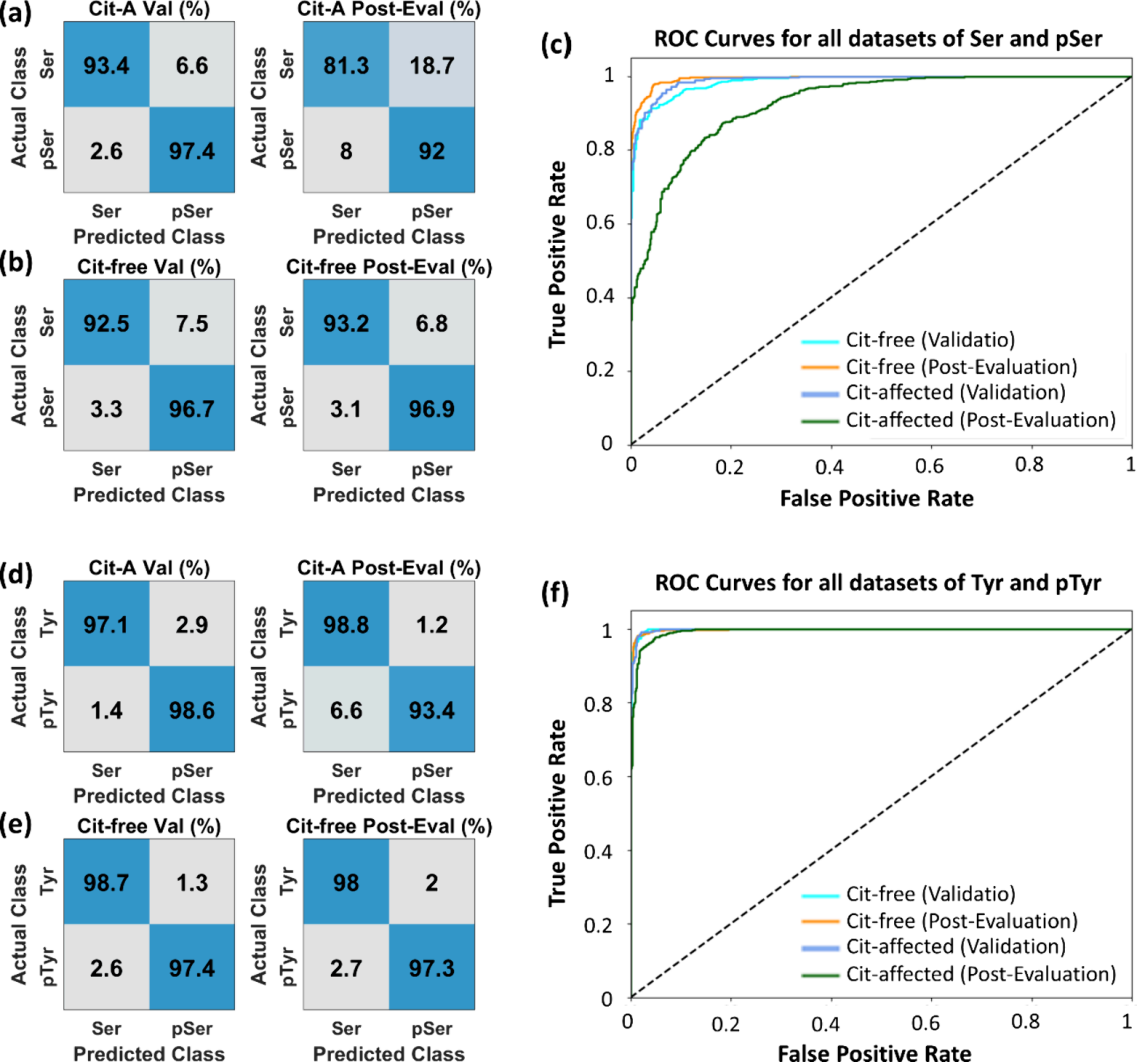
Performance metrics of the 1D-CNN on the identification of Ser from pSer and Tyr from pTyr. The confusion matrices show the classification accuracies at validation (Val) and post-evaluation (Post-Eval) stages of (a,d) citrate-affected spectra (Cit-A) and (b,e) citrate-free spectra­(Cit-free), respectively. (c,f) Corresponding ROC curve on citrate-affected and citrate-free spectra on the validation and post-evaluation sets.

To interpret the 1D-CNN result and gain insight of the data analysis, we combined the histogram of SM-SERS peak occurrence frequencies of all citrate-free data sets with the Gradient-Weighted Class Activation Mapping (Grad-CAM).^38,41,42^ The latter elucidates the decision-making process of neural networks by highlighting more discriminative spectral regions. In Figure 5, we present the normalized Grad-CAM feature weights of Ser/pSer, Tyr/pTyr (colored curves) alongside the SM-SERS peak occurrence frequencies (gray histogram) within the 500–1650 cm^–1^ spectral regions. In fact, the distinct differences in the high Grad-CAM features could correspond to either inherent molecular vibrations of the two molecules or just data differences without the corresponding vibrational modes. A good example of the latter is the high pTyr Grad-CAM feature at around 1027 cm^–1^ that does not correspond to a high SM-SERS peak occurrence frequency in Figure 5d. Therefore, only those Grad-CAM regions overlapping with a high SM-SERS peak occurrence frequency were confirmed to represent molecular structural differences and can be assigned to a certain vibrational mode. Due to the lack of a comprehensive spectral database for tyrosine and serine phosphorylation, some frequently occurring peaks may not be assigned.

**Figure 5:**
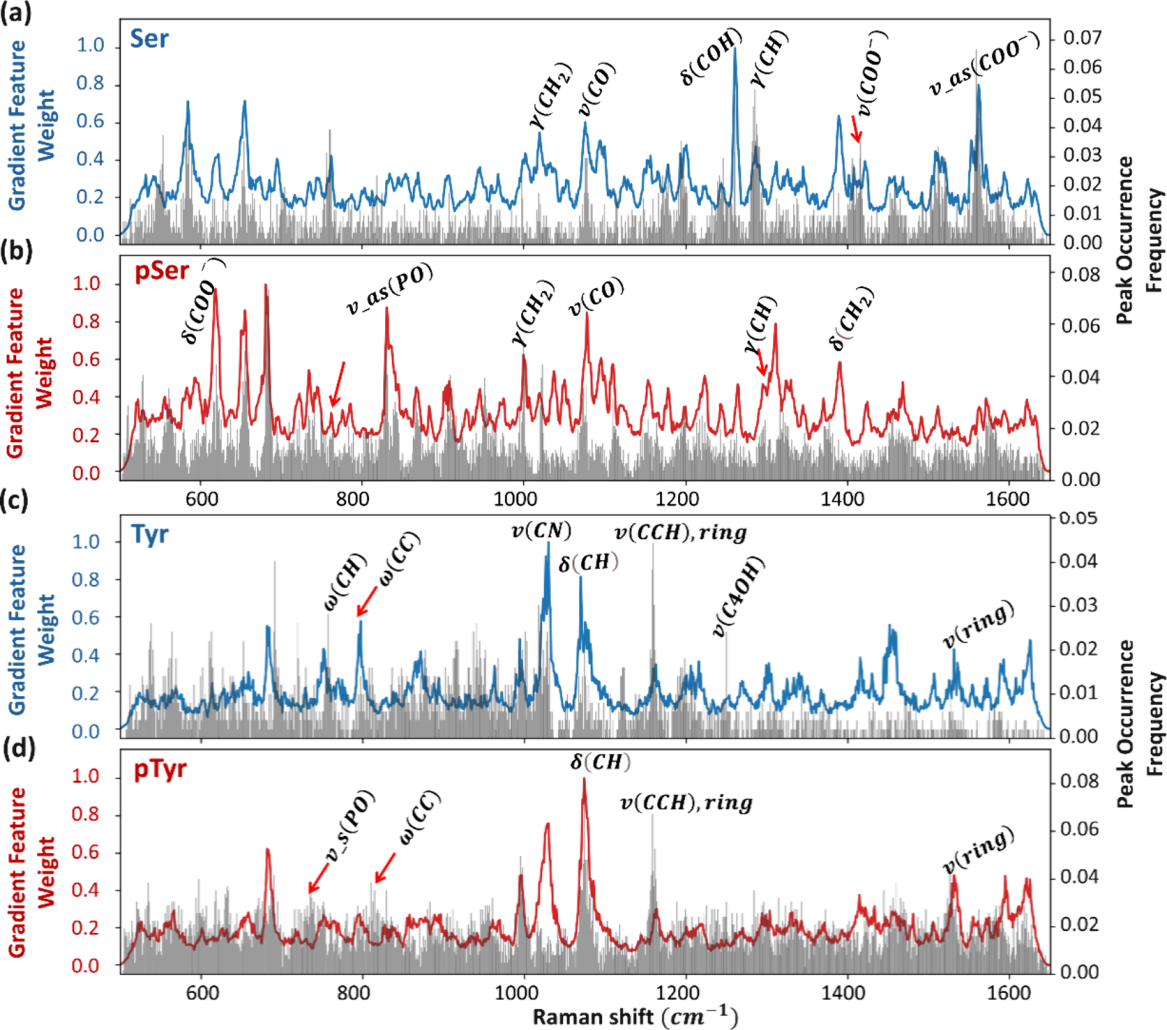
Normalized Grad-CAM feature weights extracted by the 1D-CNN model with a histogram of peak occurrence frequency of entire citrate-free data set. The blue curve in (a) represents the 1D Grad-CAM feature weights for Ser, while the red curve in (b) shows those for pSer. Similarly, the blue curve in (c) corresponds to Tyr, and the red curve in (d) corresponds to pTyr. Gray spikes in each panel indicate the peak occurrence frequencies of the corresponding molecules.

For Ser and pSer in Figure 5a,b, we observed the most significant spectral regions within the ranges 610–620 cm^–1^, 760–770 cm^–1^, 820–830 cm^–1^, 1000–1010 cm^–1^, 1090–1100 cm^–1^, 1240–1250 cm^–1^, 1300–1310 cm^–1^, 1320–1330 cm^–1^, 1420–1430 cm^–1^, and 1570–1580 cm^–1^. We assign peaks based on high occurrence frequencies, considering a Raman shift tolerance of 10 cm^–1^. The primary reliance on peak occurrence frequency for assignment stems from its direct correlation to structural differences. In contrast, the 1D-gradient feature map, derived from data differences, focuses on unique features for robust and accurate binary classification (e.g., Ser from pSer). This gradient map may also incorporate Raman peaks if they positively contribute to distinguishing the two classes; in that case, they can be assigned to a certain vibrational mode.

While pSer partly shares some spectral features with Ser, it also introduces a distinct vibrational band due to the presence of the phosphate group. For instance, both Ser and pSer exhibit C=O stretching at 1097 cm^–1^ and CH_2_ bending modes at 1361 cm^–1^. Furthermore, relative shift in shared vibrational modes due to molecular structural differences, e.g., the out-of-plane bending mode of CH2 at 1004 cm^–1^, and 1021 cm^–1^ for pSer, and Ser, respectively. Additionally, phosphorylation induces unique peaks, such as the P–O asymmetric stretching at 829 cm^–1^, which is a direct consequence of the added phosphate group,^43^ consistent with literature findings summarized in Table 1.

**Table 1:** SERS Bands with SM-SERS Peak Occurrence Frequencies as Shown in Figure 5

**Ser and pSer Band (cm** ^**‑1**^ **)**	**Molecule**	**Vibration Mode**	**Reference**
611	pSer	δ(*COO* ^–^)	^44,45^
770	Ser	γ(*COO* ^–^)	^44^
829	pSer	*v*_*as*(*PO*)	^43^
1004	pSer	γ(*CH*_2_)	^44–46^
1021	Ser	γ(*CH*_2_)	^44,45^
1097	Ser, pSer	*v*(*CO*)	^44,46^
1245	Ser	δ(*COH*)	^44^
1301	*Ser*	γ(*CH*)	^44,45^
1324	*pSer*	γ(*CH*)	^44,45^
1423	Ser	*v*(*COO*^–^)	^44^
1571	Ser	*v*_*as*(*COO*^–^)	^44^

**Tyr and pTyr Band (cm-1)**	**Molecule**	**Vibration Mode**	**Reference**
742	Tyr	*w*(*CH*)	^46^
759	pTyr	*v*_*s*(*PO*)	^43^
800	Tyr, pTyr	ω(*CC*)	^47^
1027	Tyr	*v*(*CN*)	^46,48^
1077	Tyr, pTyr	δ(*CH*)	^47^
1140	Tyr, pTyr	*v*(*CCH*), *ring*	^48^
1259	Tyr	*v*(*C*4*OH*)	^48^
1566	Tyr, pTyr	*v*(*ring*)	^48^

In the case of Tyr and pTyr in Figure 5c,d, prominent vibrational modes were identified in the ranges of 740–760 cm^–1^, 800–810 cm^–1^, 1020–1030 cm^–1^, 1070–1080 cm^–1^, 1140–1150 cm^–1^, 1250–1260 cm^–1^, and 1560–1570 cm^–1^. We observed a common feature for both molecules, including wagging of CC (800 cm^–1^), bending of CH (1077 cm^–1^), and ring stretching (1140 cm^–1^ and 1566 cm^–1^). Uniquely, asymmetric PO stretching at 759 cm^–1^ was observed, which is typically associated with pTyr, while the C4OH stretching at 1259 cm^–1^ characterizes Tyr. A comprehensive assignment of these vibrational modes is provided in Table 1.

In summary, we detected single-molecule SERS spectra of Ser, Tyr, and their phosphorylation with analyte molecules occupied only 1.23% of the particle surface using the particle-in-pore sensor, effectively excluding the citrate-affected spectra using a k-means clustering algorithm and distinguished single amino acids from their phosphorylation using a deep learning model. Our 1D-CNN model achieved accuracies of over 95% in distinguishing Ser from pSer and 97% in distinguishing Tyr from pTyr. This work demonstrates the SM-SERS identification of serine and tyrosine from their phosphorylated forms. Achieving a higher amino acid differentiation from their PTMs at a single molecule level lays the foundation for further study of peptide and protein phosphorylation. Consequently, AI-enhanced SERS will transform fields such as biochemistry, molecular biology, proteomics, genomics, and medicine by enabling precise molecular characterization at single-molecule sensitivity.

Our findings highlight deep learning analysis of SM-SERS spectra to detect PTMs. Manual analysis of thousands of single-molecule SERS spectra is impractical and susceptible to bias; conversely, deep learning makes this task automated and avoids human intervention. Notably, in deep learning, a large amount of data is required for training, validation, and post-evaluating the 1D-CNN model. The Brownian motion of molecules in the hot spot allows us to collect a large amount of original data without the need for augmentation in SERS spectra that may lead to false spectra generation. This is because the SM-SERS spectra have only one dimension, so augmenting by shifting or adding noise may introduce fake spectra.^49^ Furthermore, the high sensitivity of SM-SERS spectra renders traditional augmentation less effective in the single molecule regime. Future research may extend this approach to peptide and protein identification and sequencing at the single molecule level. The current model can be further customized for peptide and protein sequence analysis. Hence peptides have a long sequence, and their phosphorylations have many amino acids in common; the PTM site might not necessarily be located in the hot spot for SERS detection. This leads to heavy overlaps in the spectra of the peptide and its PTMs. Therefore, we may need deeper and more complex tuning of the model architecture to differentiate peptide spectra effectively.

## Supplementary Material





## Data Availability

Codes are available in the github page: https://github.com/MulusewWondie/Single-molecule-Phosphorylation-Identification-.

## Notes

Details on materials and nanofabrication; SERS signal preprocessing; K-means-based citrate removal; the data set preparation and detailed description and implementation of 1D-CNN (PDF)
Transparent Peer Review report available (PDF)
