# Supplementary material for: Single-Molecule SERS Detection of Phosphorylation in Serine and Tyrosine Using Deep Learning-Assisted Plasmonic Nanopore
Source: J Phys Chem Lett. 2025 Aug 8;16(33):8418–26. doi: 10.1021/acs.jpclett.5c01753 (PMC12376110; doi:10.1021/acs.jpclett.5c01753)
Supplement: Supplementary file 1 [file jz5c01753_si_001.pdf]

# Supporting Information

## Single-Molecule SERS Detection of Phosphorylation in Serine and Tyrosine Using Deep Learning-Assisted Plasmonic Nanopore

Mulusew W. Yaltaye[1,2,3], Yingqi Zhao[1, *], Kuo Zhan [1,3] Eva Bozo [1], Pei-Lin Xin [1,3], Vahid Farrahi [1,3,4], Francesco De Angelis[5], and Jian-An Huang [1,2,3,*]

[1] Research Unit of Health Sciences and Technology, Faculty of Medicine, University of Oulu, Aapistie 5 A, 90220 Oulu, Finland.

[2] Research Unit of Disease Networks, Faculty of Biochemistry and Molecular Medicine, University of Oulu, Aapistie 5 A, 90220 Oulu, Finland.

[3] Biocenter Oulu, University of Oulu, Aapistie 5 A, 90220 Oulu, Finland.

[4] Institute for Sport and Sport Science, TU Dortmund University, Dortmund, Germany.

[5] Istituto Italiano di Tecnologia, Via Morego 30, 16163, Genoa, Italy.

*Email: jianan.huang@oulu.fi, yingqi.zhao@oulu.fi

**Contents**

Materials....................1
Nanopore Fabrication....................1
Attachment of amino acid on AuNPs....................1
Calculation of amino acids concentration and particle surface coverage....................1
SERS Signal Pre-processing....................2
Feature of single molecule spectra....................4
K-means-based citrate removal....................5
Dataset preparation....................7
1D-CNN model description and implementation....................9
References....................13

**Materials**

Nonfunctionalized gold nanoparticles (AuNPs) with average particle size of 50 nm were from Sigma (753645-25ML, concentration of $3.5 \times 10^{10}$ particles/mL). The synthetic amino acids were ordered from Biomatik. SYLGARD™ 184 Silicone Elastomer Kit was used for Polydimethylsiloxane (PDMS) microfluidic channel fabrication. Silicon wafers with 100nm SiN membranes coated on the surface were purchased from MicroChemicals GmbH.

**Nanopore Fabrication**

The gold nanopores were fabricated on commercial SiN membranes supported on silicon. The size and thickness of the SiN window was 1 × 1mm and 100nm respectively. After sputtering a 2 nm titanium and 100 nm gold layer on the front side and 2nm gold layer at back side of the SiN membrane, Focused Ion Beam (FIB) milling (FEI Helios DualBeam) from the back side of the membrane to create nanopores with 200nm diameter. Scanning electron microscope was used to characterize nanopore size and morphology. Then the nanopore samples were embedded in a microfluidic chamber made from PDMS.

**Attachment of amino acid on AuNPs**

All the amino acids used in the measurements were attached physically on φ50 nm gold nanoparticles (AuNPs). In the final solution for Raman measurements, the concentration of AuNPs and the salt concentration were $1.3 \times 10^{10}$ particles per mL and 5% of PBS buffer. Amino acid stock solutions were mixed with gold nanoparticles and PBS buffer. Before Raman measurement, the mixture were kept in a refrigerator at 4 °C for two days to allow the adsorption of analytes on AuNP. The concentrations of amino acids in the final solution were calculated according to previous literature to ensure the number of molecules adsorbed on each AuNP is far from forming a monolayer, therefore in the particle-in-pore system only one molecule occupies the hot spot and generates single molecule SERS signal. The details of the concentration calculation are in Supporting Information **Table S1**.

**Calculation of amino acids concentration and particle surface coverage**

The concentration of AA required to achieve monolayers on the AuNPs' surfaces was determined by empirical values of maximum solvent accessibilities of residues in proteins found in the literature [1] and is shown in **Table S1**. The amount of amino acids molecules required to form a monolayer on a single AuNP was calculated, then multiplied by a factor 1/80 to further lower the percentage of molecule coverage on the AuNP surface. The selection of this factor is a balance between low molecule numbers needed to generate a single molecule signal and guarantee sufficient trapping events in the

flow-through SERS measurement. Then we determined the final molecule concentration taking account of the AuNP concentration of $1.3 \times 10^{10}$ $mL^{-1}$. The final concentration of amino acids is listed in **Table S1** below:

***Table S1***. *Surface area of amino acids, the number of molecules per AuNP and final concentrations calculated for each molecule to have a sub monolayer of molecules on the AuNP surface.*

| Amino Acids | Maximum accessible surface area ($Å^2$) [1] | Number of molecules per AuNP to form Monolayer | Number of molecules per AuNP | Final concentration (nM) |
|---|---|---|---|---|
| Ser | 143 | 5490 | 68 | 1.40625 |
| pSer | 143 | 5490 | 68 | 1.40625 |
| Tyr | 255 | 3078 | 38 | 0.7985 |
| pTyr | 255 | 3078 | 38 | 0.7985 |

**SERS Signal Pre-processing**

To prepare the SERS signals for the 1D-CNN analysis, we conducted extensive signal processing to ensure a consistent dataset. This included cosmic ray removal using median filtering algorithm followed by Asymmetric least square baseline correction. Subsequently, the Savitzky-Golay [2] smoothing algorithm and min-max normalization are applied to further remove noise and normalize the SERS signals, respectively. Since we measured the spectra in time series, an empty nanopore generated only noise basline in the absence of trapped particles. Thus, we good SERS peak characterstic features signal-to-noise ratio (SNR) to select the most significant spectra from the status when the particle was trapped. Due to the weak single-molecule signals, a threshold of SNR ≥ 2.5 is applied to select spectra correspoding to trapping event and preclude those belonging to the empty nanopore status details.

**Cosmic ray removal.** Before feeding the data into the CNN model, we conducted extensive SERS spectra processing to build a consistent dataset. SERS spectra affected by cosmic rays as shown in Figure S1(a), we observe high intense artifacts so called "spikes", a sudden sharp peak that appeared due to imperfection of electronic circuit and abnormal behavior of detector.[3] These "spikes" neither characterize the molecule nor preserve its integrity, as they degrade organic compounds and thereby diminish and distort the detectable Raman signatures.[4] So, it is important to remove these defects to

prevent miss-identification of the signal of interest.[5] We apply median filtering to remove cosmic rays in MATLAB, result a cosmic removed spectrum in Figure S1(b).

**Baseline correction.** The Raman spectrum is significantly affected by a baseline signal which is mainly caused by fluorescence during Raman measurement. Fluorescence backgrounds are several orders of Raman signals [6] and they should be removed or suppressed. We used Asymmetric Least squares (ALS) algorithms. We chose these baseline correction strategies based on two criteria: their effectiveness in removing the baseline signals and their ability to minimize distortion in SERS spectra.

**Smoothing.** Savitzky-Golay(S-G) smoothing algorithm is applied to remove noises from the measured spectra. It is one of the widely used smoothing techniques [7,8] in analytical chemistry to improve spectral resolution by filtering high-frequency noise. The algorithm works by polynomial fitting [9] to subsets of adjacent data points through a least square regression method. We considered the window size of 9 and polynomial degree fitting of 3. The most significant part of this algorithm is that it preserves features like width, height, peak position, and the shape of the Raman spectra, [9] thereby minimizing distortion from the original spectra. The Savitzky -Golay technique paved an effective way of denoising Raman spectra while retaining their original shape, enabling improved resolution for deeper insights into molecular vibration and bond structures.

**Normalization.** Subsequently, we applied min-max normalization to Raman spectra. Normalization is crucial for spectroscopic data due to the dependency of the SERS signals on the enhancement techniques, optical setup, and measurement parameters. Normalization ensures the consistent dataset for deep learning models.

**Thresholding.** We used peak feature characteristics including prominence, peak heigh, and peak width and signal-to-noise ratio (SNR) thresholding to preclude noisy signals from our measurement. SNR of 2.5 is considered to select the most significant SERS spectra that characterizes the molecules better.

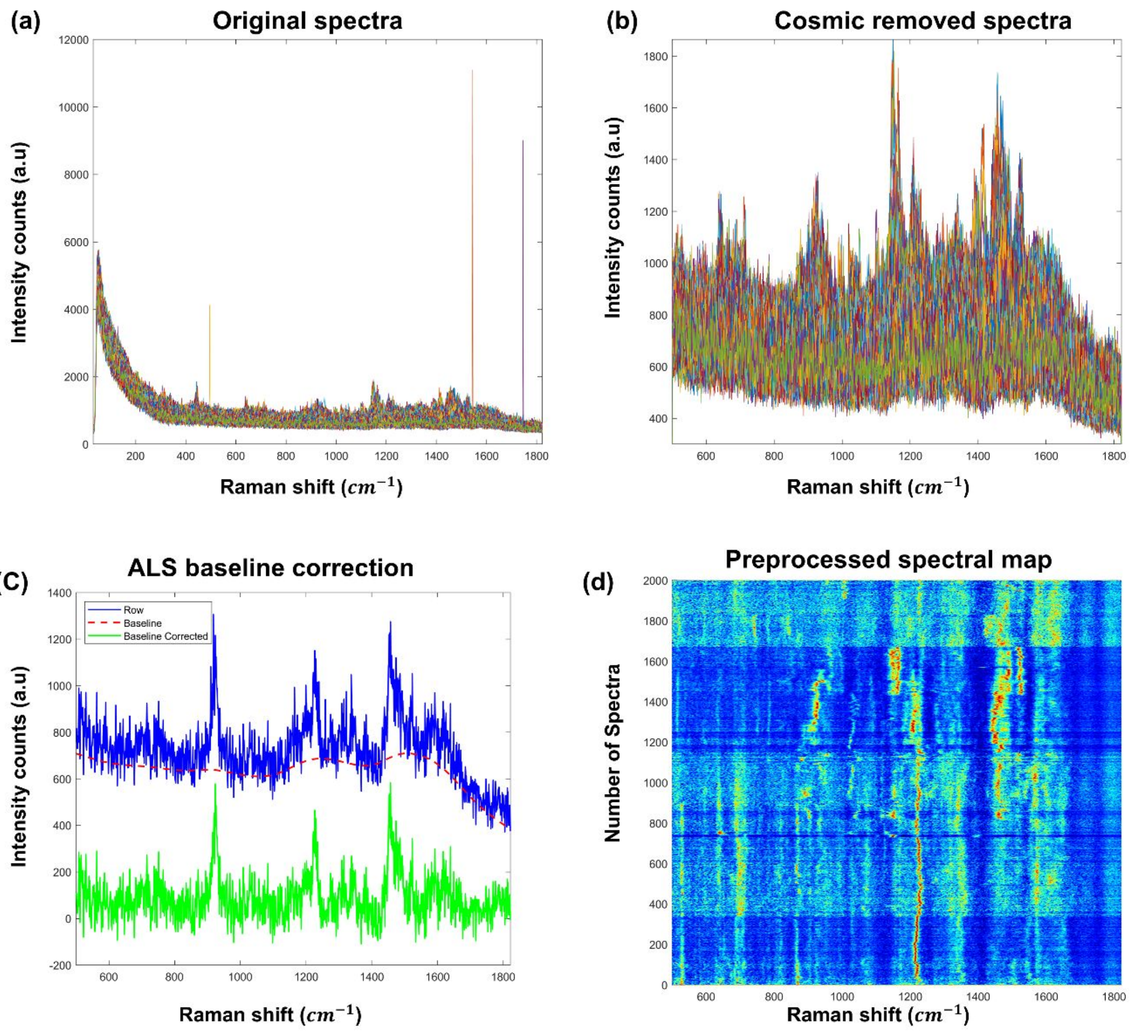


***Figure S1**. (a) Raw SERS spectra dominated by cosmic rays. (b) Spectra after cosmic ray removal using median filtering. (c) Implementation of the asymmetric least squares algorithm for baseline correction on a single spectrum: the blue line represents the original signal, the red curve shows the estimated baseline, and the green line is the baseline-corrected signal. (d) Preprocessed spectral map after smoothing and normalization.*

## Feature of single molecule spectra

Single-molecule spectra are characterized by distinct spectral fluctuations that arise from the dynamic the Brownian motion of the nanoparticles and the adsorption and desorption of single molecule in the hot spot. These fluctuations reflect changes in molecular orientation, conformational states, and transient interactions. As shown in Figure S2, the intensity and position of SM-SERS peaks vary significantly over time. To address this spectral variability, we employed peak occurrence frequency analysis, which provides a robust generalization of the underlying spectral features.

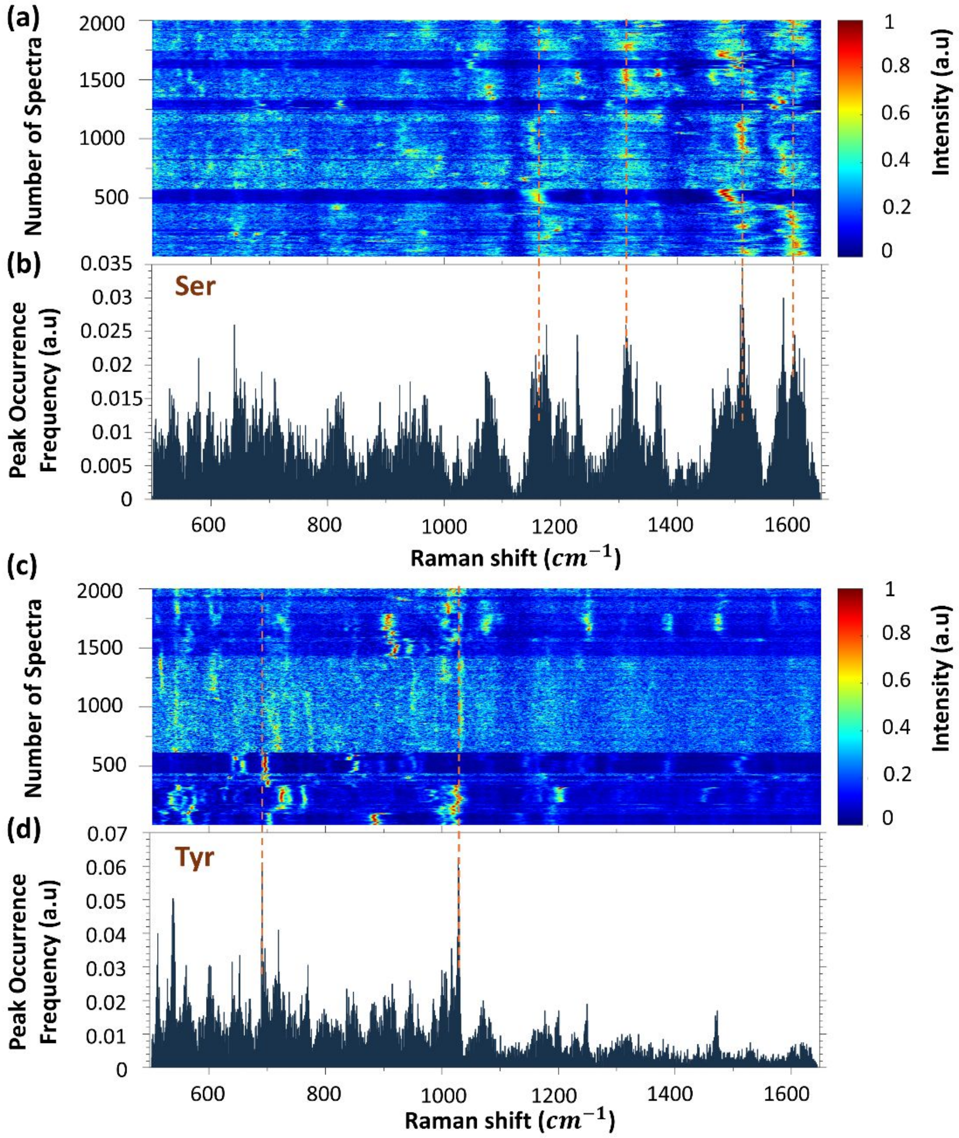


***Figure S2***. *(a) Fluctuating single-molecule SERS spectra of Ser, with the red line indicating the most frequently occurring Raman shift and the color bar showing normalized signal intensity (a.u.). (b) Peak occurrence frequency of Ser. (c) Fluctuating single-molecule SERS spectra of Tyr. (d) Peak occurrence frequency of Tyr.*

**K-means-based citrate removal**

The k-means algorithm, a widely recognized unsupervised learning method for clustering in pattern recognition and machine learning, is often influenced by initializations and requires a predefined number of clusters. [10] In our study, we determined the number of clusters based on our prior knowledge of the dataset. We further justified the number of clusters using the elbow method,[11] a common technique for identifying the optimal cluster number through manual identification of "elbow points" on the visualization curve as shown in **Figure S3**. We used the default parameters for the kmeans function were used: a maximum of 100 iterations (MaxIter), the squared Euclidean

distance metric ('sqeuclidean'), the 'singleton' option for handling empty clusters (EmptyAction), and a single replication (the number of clustering repetitions with new initial centroid positions was set 10). For implementation of principal component analysis for dimensionality reduction, we used 30 numbers of PCA components. The t-SNE function was executed using its default parameters: the Euclidean distance metric, a perplexity of 30 (representing the effective number of local neighbors for each point), a learning rate of 500, and a maximum of 1000 optimization iterations.

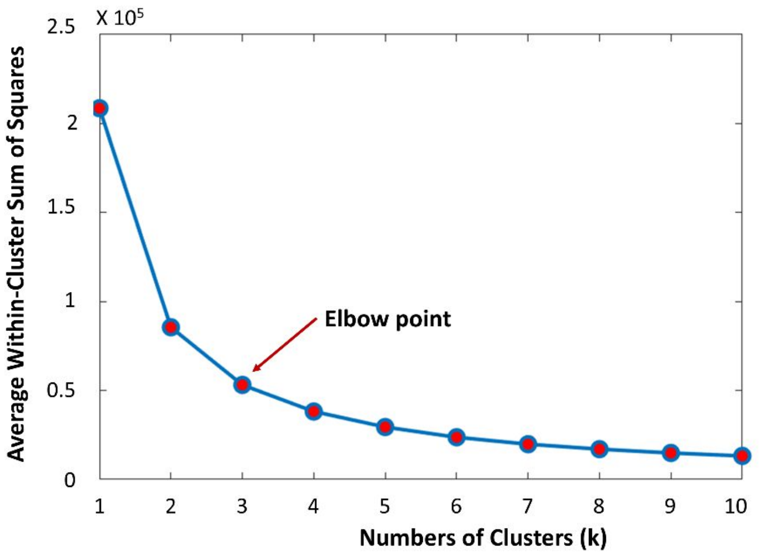


***Figure S3**. Elbow Method for determining the optimal number of clusters (k) on Ser, pSer, and Citrate datasets. The plot shows the average within-cluster sum of squares (AWCSS) as a function of number of clusters (k). The "elbow point"  the red arrow pointing the optimal number of elbow points.*

Following the k-means clustering, we identified the cluster predominantly composed of citrate spectra using MATLAB function so-called “mode”. This cluster was then established as a reference to identify and filter out citrate-contaminated spectra. By iteratively assessing each of the target molecule spectrum using MATLAB function “find”, we determined if a spectrum was assigned to the citrate reference cluster. If so, the spectrum was labeled "citrate-affected" and removed from the dataset. Notably, the citrate-affected spectrum might still contain spectral peaks from the target molecule; however, it was removed because its overall signature was dominated by or highly interfered by citrate peaks. This technique resulted in an improvement in the post-evaluation performance of our one-dimensional convolutional neural network (1D-CNN) model after the citrate-affected spectra was excluded.

For distinct visualization of the different clusters, we used principal component analysis (PCA) followed by t-SNE to visualize the spectral clustering outcomes as distinct groups in a two-dimensional

space. The relative number of datapoints in each cluster presented in **Figure S4.** Our original dataset, which includes data for an amino acid, its phosphorylation, and citrate, was used to provide ground truth labels for the clustering process. For the two-class clusters, the extracted pure spectra were then used as a new ground truth label.

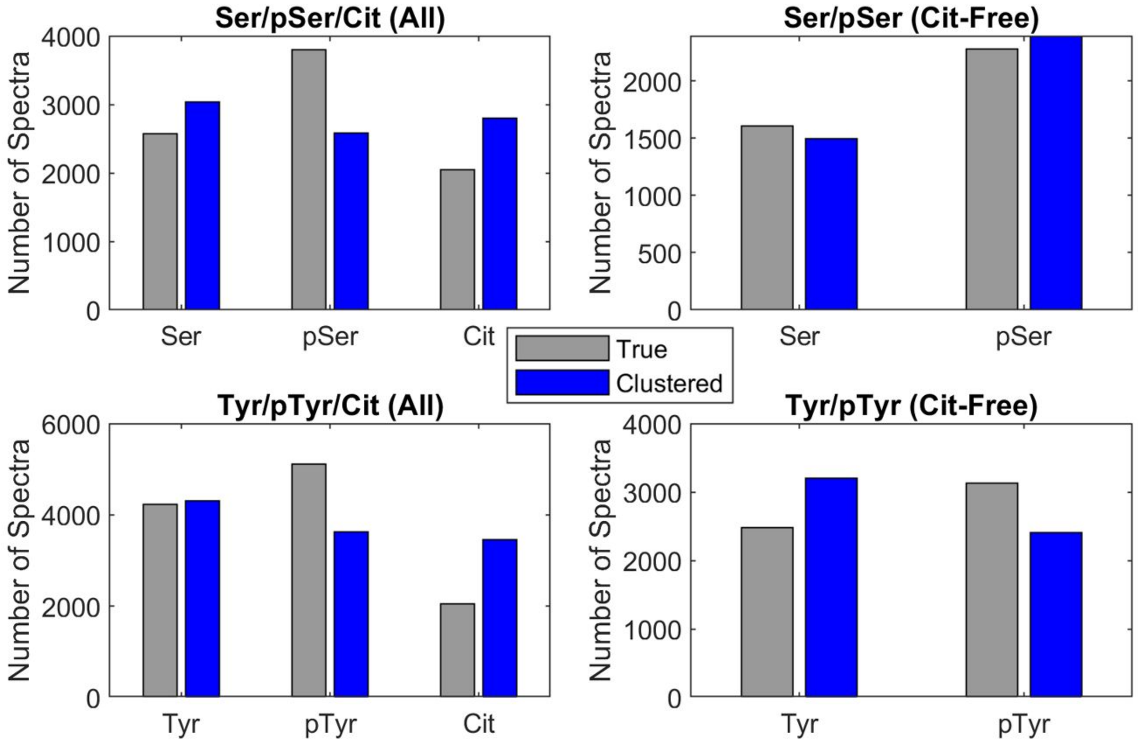


***Figure S4***. *The bar graph demonstrates the relative counts of SERS spectra in each cluster, with the gray bars indicating the ground truth labels and the blue bars representing the number of spectra (data points) after k-means clustering.*

**Dataset preparation**

After processing a SM-SERS spectra, citrate-affected and citrate-free datasets were constructed and split into training, validation, and post-evaluation sets for model development. The citrate-free dataset is only 60% of the original (citrate-affected) dataset, i.e. 40% of the original datasets are affected by citrate.

**Table S2**. Single molecule SERS dataset for validating and post-evaluating the 1D-CNN model.

| Cit-affected | Total | Training Set | Validation Set | Post-evaluation |
|---|---|---|---|---|
| Ser | 2572 | 1444 | 619 | 509 |
| pSer | 3803 | 2128 | 912 | 763 |
| Tyr | 4231 | 2365 | 1013 | 853 |

| | | | | |
|---|---|---|---|---|
| pTyr | 5108 | 2847 | 1220 | 1041 |
| **Cit-free** | | | | |
| Ser | 1605 | 897 | 385 | 321 |
| pSer | 2281 | 1277 | 548 | 456 |
| Tyr | 2484 | 1391 | 596 | 497 |
| pTyr | 3125 | 1750 | 750 | 625 |

**Spectral profile of removed spectra**: We compared the pure citrate signals with the citrate-affected ones in Figure S5 below. Notably, these signals share a pure citrate peak at 1070 $cm^{-1}$, which was assigned to the $\nu_{CO}$ mode of citrate. We calculated the Euclidean distance between the mean spectrum of pure citrate and the mean of the citrate-affected spectra precluded by k-means based clustering. The Euclidean distance between was 2.6042 for Ser/pSer and 2.8853 for Tyr/pTyr. These low distance values, combined with the observed spectral overlap, confirm that the spectra identified as citrate-affected share a high degree of similarity with the pure citrate spectra. The shaded region represents the standard deviation of the individual spectra from the mean.

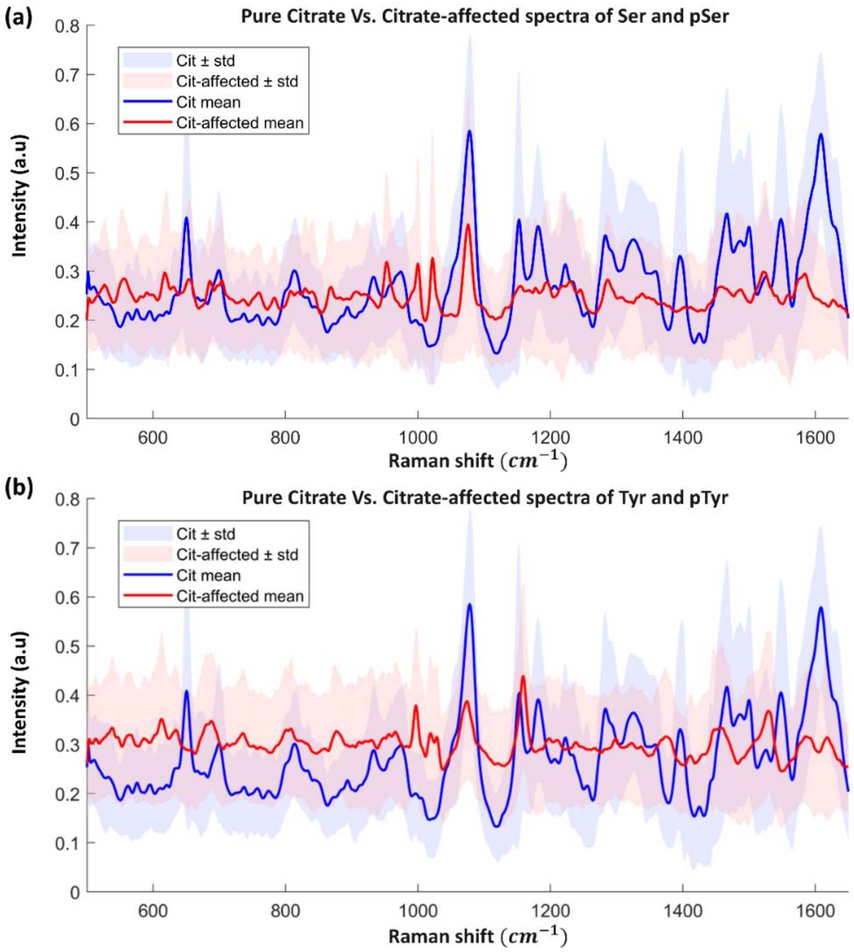


***Figure S5.*** *Average spectra of (a) citrate and citrate-affected Ser/pSer and (b) citrate and citrate-affected Tyr/pTyr. The shaded regions represent the standard deviation at each wavenumber.*

**1D-CNN model description and implementation**

The model architecture consists of an input, three convolutional blocks, a flattening layer, two fully connected blocks (dense blocks), and an output layer. Each convolutional block consists of two convolutional layers, two batch normalization (BN) layers, the maxpooling layer, and dropout layers. We started a convolutional layer with 16 filters and proceeded to a convolutional layer having 64 filters by doubling each time. Batch normalization accelerates convergence,[12] while max-pooling reduces spatial dimensions and dropout prevents overfitting. [13] The flattening layer [14] is used to transform multiple feature maps produced by the convolutional layers into 1D vector. The rectifier linear unit (ReLU) activation function introduces non-linearity to the model and mitigates the vanishing gradient. [15,16] The fully connected (Dense layer) basically do the final decision after the convolutional and pooling layers extract features, which should learn the global patterns in their input feature spaces to classify them. Finally, the Sigmoid activation function in the output layer is used to convert raw score

outputs into a probability distribution over the binary classes. The model architecture for binary classification of Ser Vs. pSer and Tyr Vs. pTyr is drawn in **Figure S6**.

The model was trained using an Adam optimizer, utilizing a kernel L2 regularization with a learning rate of $e^{-4}$. Regularization helps in mitigating overfitting by penalizing overly complex models and encouraging simpler ones, thereby improving a deep learning model's ability to generalize to data points. [17,18] The performance metrics including loss, accuracy, the area under the ROC (receiver operating characteristics) curves were used to evaluate the performance of the model during the training, validation and post-evaluation phases. To prevent overfitting, dropout regularization of 30% and 50% added after each convolutional block and dense layer respectively. An Early stopping with patience of 10 epoches implemented to monitor validation accuracy, the model will terminate at its best performance if there is no improvement after 10 epochs. The performance of the model during training is presented in **Figure S7** on Ser vs. pSer identification.

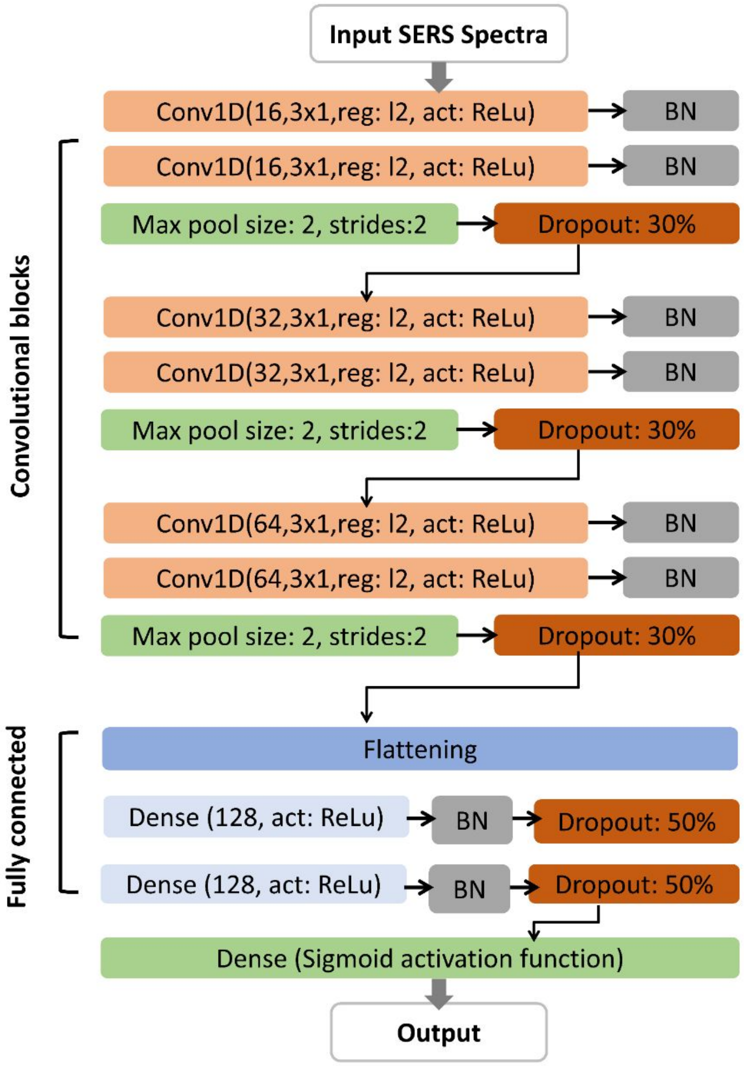

***Figure S6.*** *The detailed architecture of the 1D-CNN model, designed for distinguishing Ser from pSer and Tyr from pTyr. The CNN architecture consists of three convolutional blocks, each comprising two convolutional layers, batch normalization, max-pooling, and dropout layers.*

**Table S3**. The detailed implementation of 1D-CNN model.

| Layers | Types | Detailed parameters |
|---|---|---|
| **Input** | Input layer | Shape = (1048,1 ) |
| **Conv #1** | Conv1d(x2), BatchNormalization (x2),Maxpooling1D,Dropout | Conv1D(16,3,1),Maxpool(2,2), Dropout(0.3),Activation(ReLU) |
| **Conv #2** | Conv1d(x2), BatchNormalization (x2),Maxpooling1D,Dropout | Conv1D(32,3,1),Maxpool(2,2), Dropout(0.3),Activation(ReLU) |
| **Conv #3** | Conv1d(x2), BatchNormalization (x2),Maxpooling1D,Dropout | Conv1D(64,3,1),Maxpool(2,2), Dropout(0.3),Activation(ReLU) |
| **Flattening** | Flatten | - |
| **Dense** | Dense, BatchNormalization, Activation, Dropout | Dense(128), Dropout(0.5) , Activation(ReLU) |
| **Dense** | Dense, BatchNormalization, Activation, Dropout | Dense(128), Dropout(0.5) ,Activation(ReLU) |
| **Output** | Dense | Output(2), Activation(Sigmoid) |

The architecture of the model for single amino acid identification: the parameters in conv1D represent (filter, kernel_size, strides), Maxpool(pool_size, strides), Dropout(rates), Dense(units). Note: we used the rectifier linear unite (ReLU) activation function in all convolutional blocks, and the sigmoid activation function is used for the output layer.

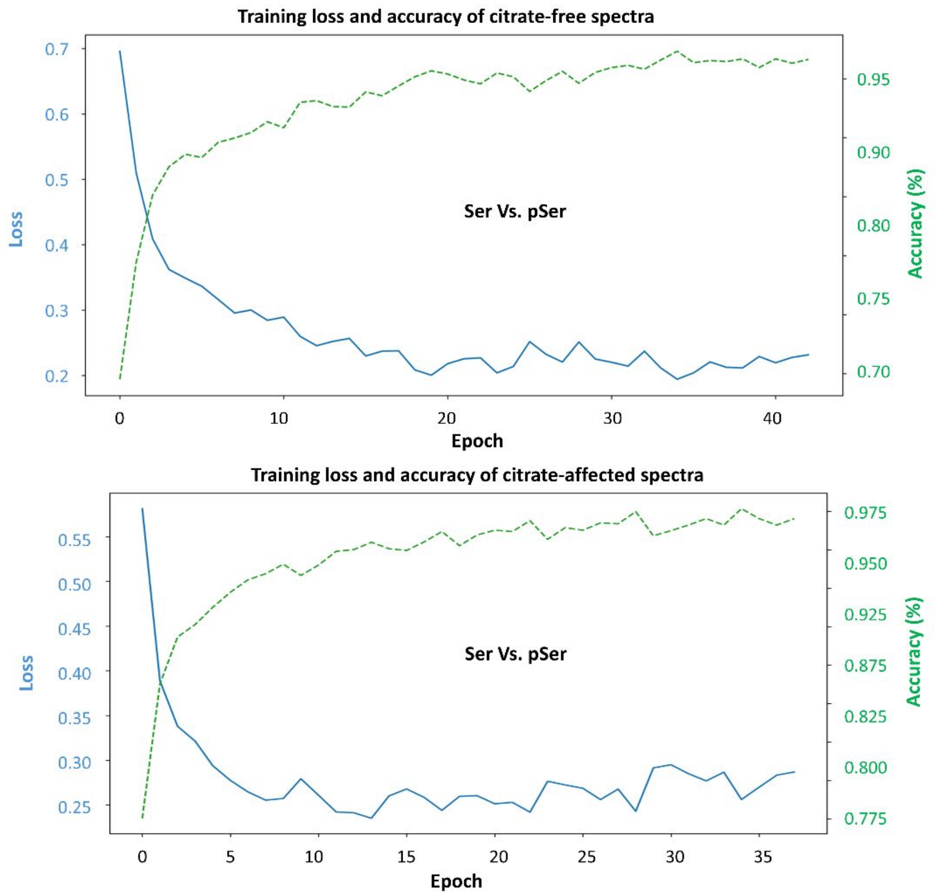


***Figure S7.*** *The performance of the model on citrate-affected and citrate-free datasets of Ser VS pSer in terms of accuracy and loss during training, showing a progressive increase in accuracy and a decrease in loss over epochs, indicating successful model convergence.*

**Table S4**: Performance of the model on the training and post-evaluation sets for both citrate-free and citrate-affected spectra.

| Dataset (Ser Vs. pSer) | AUC (%) | Precision (%) | Recall (%) |
|---|---|---|---|
| Cit-free Training | 99.08 | 97.89 | 97.32 |
| Cit-free post-evaluation | 97.15 | 99.09 | 94.86 |
| Cit-affected Training | 98.89 | 94.98 | 97.59 |
| Cit-affected post-evaluation | 86.24 | 95.04 | 91.01 |
| Dataset (Tyr Vs. pTyr) | AUC (%) | Precision (%) | Recall (%) |
| Cit-free Training | 99.74 | 98.19 | 97.52 |
| Cit-free post-evaluation | 98.71 | 98.71 | 98.08 |
| Cit-affected Training | 99.48 | 96.98 | 96.90 |

| | | | |
|---|---|---|---|
| Cit-affected post-evaluation | 92.32 | 97.76 | 99.38 |

**References**


(1) Tien, M. Z.; Meyer, A. G.; Sydykova, D. K.; Spielman, S. J.; Wilke, C. O. Maximum Allowed Solvent Accessibilites of Residues in Proteins. *PLoS One* **2013**, *8* (11), e80635. https://doi.org/10.1371/journal.pone.0080635.

(2) Savitzky, Abraham.; Golay, M. J. E. Smoothing and Differentiation of Data by Simplified Least Squares Procedures. *Anal Chem* **1964**, *36* (8), 1627–1639. https://doi.org/10.1021/ac60214a047.

(3) Ehrentreich, F.; Summchen, L. Spike Removal and Denoising of Raman Spectra by Wavelet Transform Methods. *Anal Chem* **2001**, *73* (17), 4364–4373. https://doi.org/10.1021/ac0013756.

(4) Dartnell, L. R.; Page, K.; Jorge-Villar, S. E.; Wright, G.; Munshi, T.; Scowen, I. J.; Ward, J. M.; Edwards, H. G. M. Destruction of Raman Biosignatures by Ionising Radiation and the

Implications for Life Detection on Mars. *Anal Bioanal Chem* **2012**, *403* (1), 131–144. https://doi.org/10.1007/s00216-012-5829-6.

(5) Zhang, L.; Henson, M. J. *A Practical Algorithm to Remove Cosmic Spikes in Raman Imaging Data for Pharmaceutical Applications*.

(6) Wei, D.; Chen, S.; Liu, Q. Review of Fluorescence Suppression Techniques in Raman Spectroscopy. *Appl Spectrosc Rev* **2015**, *50* (5), 387–406. https://doi.org/10.1080/05704928.2014.999936.

(7) Brown, C. D.; Vega-Montoto, L.; Wentzell, P. D. Derivative Preprocessing and Optimal Corrections for Baseline Drift in Multivariate Calibration. *Appl Spectrosc* **2000**, *54* (7), 1055–1068. https://doi.org/10.1366/0003702001950571.

(8) Zimmermann, B.; Kohler, A. Optimizing Savitzky-Golay Parameters for Improving Spectral Resolution and Quantification in Infrared Spectroscopy. In *Applied Spectroscopy*; 2013; Vol. 67, pp 892–902. https://doi.org/10.1366/12-06723.

(9) Guiñón, J. L.; Ortega, E.; Guiñón, J. L.; García-Antón, J.; Pérez-Herranz, V. *Moving Average and Savitzki-Golay Smoothing Filters Using Mathcad*; 2007. https://www.researchgate.net/publication/228407245.

(10) Sinaga, K. P.; Yang, M.-S. Unsupervised K-Means Clustering Algorithm. *IEEE Access* **2020**, *8*, 80716–80727. https://doi.org/10.1109/ACCESS.2020.2988796.

(11) Shi, C.; Wei, B.; Wei, S.; Wang, W.; Liu, H.; Liu, J. A Quantitative Discriminant Method of Elbow Point for the Optimal Number of Clusters in Clustering Algorithm. *EURASIP J Wirel Commun Netw* **2021**, *2021* (1), 31. https://doi.org/10.1186/s13638-021-01910-w.

(12) Bjorck, J.; Gomes, C.; Selman, B.; Weinberger, K. Q. *Understanding Batch Normalization*.

(13) Yao, L.; Xu, G.; Zhao, F. Pooling Method On PCNN in Convolutional Neural Network. *J Phys Conf Ser* **2020**, *1486* (2), 022026. https://doi.org/10.1088/1742-6596/1486/2/022026.

(14) Chen, C.-C.; Liu, Z.; Yang, G.; Wu, C.-C.; Ye, Q. An Improved Fault Diagnosis Using 1D-Convolutional Neural Network Model. *Electronics (Basel)* **2020**, *10* (1), 59. https://doi.org/10.3390/electronics10010059.

(15) Lau, M. M.; Hann Lim, K. Review of Adaptive Activation Function in Deep Neural Network. In *2018 IEEE-EMBS Conference on Biomedical Engineering and Sciences (IECBES)*; IEEE, 2018; pp 686–690. https://doi.org/10.1109/IECBES.2018.8626714.

(16) Yu, Y.; Adu, K.; Tashi, N.; Anokye, P.; Wang, X.; Ayidzoe, M. A. RMAF: Relu-Memristor-Like Activation Function for Deep Learning. *IEEE Access* **2020**, *8*, 72727–72741. https://doi.org/10.1109/ACCESS.2020.2987829.

(17) Domingos, P. A Few Useful Things to Know about Machine Learning. *Commun ACM* **2012**, *55* (10), 78–87. https://doi.org/10.1145/2347736.2347755.

(18) Gygi, J. P.; Kleinstein, S. H.; Guan, L. Predictive Overfitting in Immunological Applications: Pitfalls and Solutions. *Hum Vaccin Immunother* **2023**, *19* (2). https://doi.org/10.1080/21645515.2023.2251830.
