# Supplementary material for: Single-Molecule SERS Detection of Phosphorylation in Serine and Tyrosine Using Deep Learning-Assisted Plasmonic Nanopore
Source: J Phys Chem Lett. 2025 Aug 8;16(33):8418–26. doi: 10.1021/acs.jpclett.5c01753 (PMC12376110; doi:10.1021/acs.jpclett.5c01753)
Supplement: Supplementary file 2 [file jz5c01753_si_002.pdf]

jz-2025-017536.R1

Name: Peer Review Information for "Single-Molecule SERS Detection of Phosphorylation in Serine and Tyrosine Using Deep Learning-Assisted Plasmonic Nanopore"

First Round of Reviewer Comments

Reviewer: 1

Comments to the Author

The manuscript proposes a complete workflow-“partical-in-pore+K-means+1D-CNN”-for single-molecule SERS identification of Ser and Tyr phosphorylation sites from citrates. The concept is innovative and the reported accuracies (>95%/97%) are impressive. The methodology is sound, the data set is sufficiently large, the Grad-CAM visualization is provided. Both the experimental and computational section have clear publication value, yet several details require clarification or supplementation. On the basis of the following points, I recommend Minor Revision.

1. Standardise language and spelling

Numerous misspellings appear in the abstract and main text:

e.g. refence, in line 37-38, page1; sensetive, in line 31-32, page2; tumer in lin 18-19, Page2.

Please perform a comprehensive proof-reading and adopt standard academic English.

2. Add missing key methodological parameters

k-means: state the rationale for the chosen k, the distance metric, and the random seed.

t-SNE: report perplexity, learning rate, and any other relevant hyper-parameters.

3. Quantify the performance of the citrate-removal step

In the Supporting Information, please provide Precision/Recall for predicting “citrate-positive” versus “true citrate” spectra, or include an ROC curve for pure-citrate versus analyte mixtures, to demonstrate that this preprocessing does not eliminate genuine signals.

4. Improve figure readability

The numerical labels in several figures are too small to be legible in print. Please enlarge the font sizes and, where appropriate, redraw the graphics as vector images to preserve clarity.

5. Reference duplication and formatting issues

References 16 and 25 cite the same article; DOI formats are inconsistent throughout the manuscript; several citations still use raw “http://” links instead of DOI links. Please standardize all references.

6. The authors have used k-means clustering and t-sne visualization to pick out the citrate-contaminant spectra, but as seen from the figures (figure 3), the distribution of the citrate spectra and the Ser/pSer (of Tyr/pTyr) spectra were mixed together without clear boundaries, so how did the author prove the reliability of citrate removal? In addition, no matter before or after the removal of citrate-contaminated spectra, the spectra of the original amino acids and the phosphorylated amino acids showed overlapping distribution, so how significant is the improvement in classifying the two kinds of spectra by citrate removal? On the other hand, have the authors checked the citrate-contaminated spectra which were picked out by the algorithm? These data should be provided for clarity and other experimental techniques to solidate the contamination should also be used for validation.

7. Please cite the recent very relevant review article (ACS Appl. Mater. Interfaces 2025, 17, 11, 16287–16379) and the research article (Digital colloid-enhanced Raman spectroscopy by single-molecule counting, Nature, 2024, 628, 771). For the pSer and pTyr, can the peak occurrence frequency also be digitalized, which will be easily counted?

The study represents a clear advance in single-molecule phosphosite detection, with a complete and convincing experimental–computational workflow. The issues raised above are chiefly clarifications or additions that do not alter the main conclusions. I therefore recommend acceptance after Minor Revision.

Reviewer: 2

Comments to the Author

General comments: The authors developed a plasmonic particle-in-pore sensor to obtain single-molecule SERS spectra of phosphorylation at Serine and Tyrosine, and the result spectral data was analyzed through deep learning method. The major advance of this manuscript is that discriminating the phosphorylation in Serine and Tyrosine with over 95% and 97% accuracies, respectively. The author should pay attention that efforts in single-molecule PTM identification have recently emerged, and the novelty claim in the paper should be cautiously positioned. This manuscript can be published on The Journal of Physical Chemistry Letters after addressing the following concerns:

1. why does the Raman peak of spectra in Figure 2a show obvious shift especially around 900 and 1200 cm-1?

2. In Figure 4d, the highlighted spectral regions from Grad-CAM do not always correspond to clear Raman peaks or chemically meaningful vibrational modes. The authors should discuss the possible implications of this.

3. While the k-means filtering step effectively reduces citrate background, it would be helpful to explicitly describe the rationale for selecting 4 clusters. Did the authors test other cluster numbers or validate the citrate-related cluster using known references?

4. What are the specific aspects of its chemical specificity of SM-SERS compared with multi-molecule detection? For the proof of chemical selectivity, there are some literatures for reference, Nat. Commun. 2024, 15: 5855.

5. The approach works well for pSer/pTyr classification, but how general is the model across different peptide sequences or environments? A short discussion on model transferability or limitations would add depth.

6. Others, Figure 2d (confusion matrix) should specify which label corresponds to which class (pSer, pTyr) more clearly; Figure 3c-d (t-SNE plots) could benefit from color bars or consistent legends to enhance readability; please clarify how “single-molecule” was determined; Please ensure that training data and model code (or a minimal implementation) are made publicly accessible;

Reviewer: 3

Comments to the Author

The manuscript presents a novel approach single-molecule SERS combined with deep learning for detecting phosphorylation in serine and tyrosine. While the integration of plasmonic nanopore sensors and machine learning is a very effective approach and potentially impactful, the manuscript suffers from several critical issues in terms of scientific rigor, clarity, and validation. In its current form, the manuscript is not suitable for publication.

1. The authors claim this is the first demonstration of single molecular SERS (SM-SERS) detection of pSer and pTyr. However, similar approaches using SERS and machine learning for PTM detection have been reported. The manuscript fails to clearly differentiate this work from prior studies (e.g., Zhao et al., Nano Lett. 2025). And also, deep learning has been widely applied in SERS analyses, and the authors should justify why current approach was chosen over other architectures.

2. The reported analyte surface coverage of 1.23% is extremely low. The authors should provide quantitative evidence (e.g., surface density measurements or other control experiments) to support the claim that meaningful SM-SERS signals can be reliably obtained under such sparse conditions. In addition, The reproducibility of the SM-SERS measurements is not addressed. How many independent experiments were conducted?

3. The use of k-means clustering for citrate signal removal is inadequately justified. How was the number of clusters selected? Were alternative clustering methods considered?

The CNN architecture is only described in the Supporting Information. The main text should include a concise but complete description of the model, including layer types, activation functions, and regularization strategies.

4. The Grad-CAM analysis is interesting but lacks rigorous interpretation. Several highlighted regions do not correspond to known vibrational modes, raising concerns about model overfitting or spurious correlations. The authors claim high accuracy (>95%) but do not report confidence intervals or statistical significance. Given the relatively small dataset (especially after citrate removal).

Here are the minor comments:

The authors should clarify whether the same CNN model was used for both Ser/pSer and Tyr/pTyr classification tasks.

The ROC curves in Figure 4 are not clearly labeled. AUC values should be reported.

The GitHub link provided is incomplete and should be verified.

Author's Response to Peer Review Comments:

Point-to-point response to the reviewers' comments

Reviewer: 1

Recommendation: This paper may be publishable, but major revision is needed; I would like to be invited to review any future revision.

Comments:

The manuscript proposes a complete workflow-"partical-in-pore+K-means+1D-CNN"-for singlemolecule SERS identification of Ser and Tyr phosphorylation sites from citrates. The concept is innovative and the reported accuracies (>95%/97%) are impressive. The methodology is sound, the data set is sufficiently large, the Grad-CAM visualization is provided. Both the experimental and computational section have clear publication value, yet several details require clarification or supplementation. On the basis of the following points, I recommend Minor Revision.

1. Standardise language and spelling Numerous misspellings appear in the abstract and main text:

   e.g. refence, in line 37-38, page1; sensetive, in line 31-32, page2; tumer in lin 18-19, Page2.

   Please perform a comprehensive proof-reading and adopt standard academic English.

Reply: Thank you for your valuable feedback on improving our manuscript. We have carefully considered your suggestions and thoroughly proofread the manuscript. This includes correcting the typos you pointed out, such as changing 'refence' to 'reference' **(page 1, line 19),** 'sensetive' to 'sensitive' **(page 2, line 20)** and 'tumer' to 'tumor' **(page 2, line 13).** And all other revisions have been highlighted in yellow, with the track changes feature for your convenience.

2. Add missing key methodological parameters k-means: state the rationale for the chosen k, the distance metric, and the random seed. t-SNE: report perplexity, learning rate, and any other relevant hyper-parameters.

Reply: Thank you for your review, which helped us significantly improve the quality and coherence of our work.

Based on our prior knowledge of the data, which included an amino acid, its phosphorylation, and citrate, we set the number of clusters to three. This was confirmed using the elbow method, as shown in **Figure S3** below. The k-means clustering allowed for an unbiased and anonymous grouping of the data into these three clusters. This was followed by a systematic segregation of the citrate-affected spectra by identifying their indices within the citrate cluster.

The **Figure S3**, parameters for the t-SNE and k-means implementations have been added to the revised supporting information **(pages 5-6)**, as quoted below.

"We used the default parameters for the kmeans function were used: a maximum of 100 iterations (MaxIter), the squared Euclidean distance metric ('sqeuclidean'), the 'singleton' option for handling empty clusters (EmptyAction), and a single replication (the number of clustering repetitions with new initial centroid positions was set 10). For implementation of principal component analysis for dimensionality reduction, we used 30 numbers of PCA components. The t-SNE function was executed using its default parameters: the Euclidean distance metric, a perplexity of 30 (representing the effective number of local neighbors for each point), a learning rate of 500, and a maximum of 1000 optimization iterations."

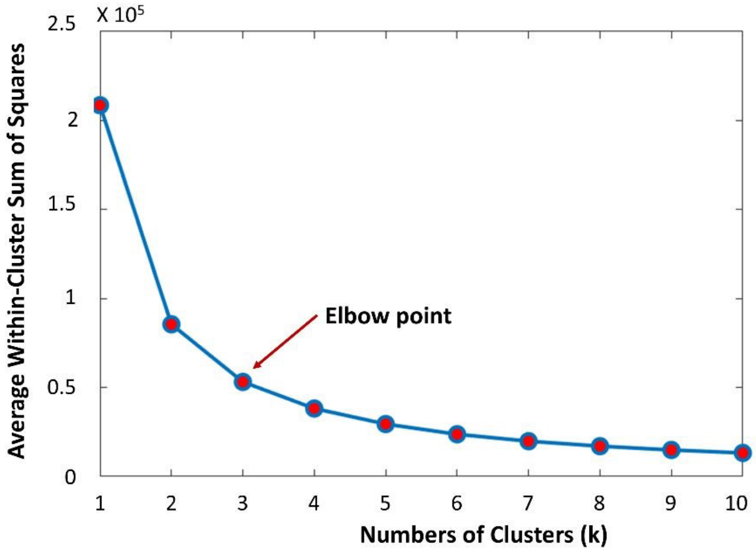

***Figure S3**. Elbow Method for determining the optimal number of clusters (k) on Ser, pSer, and Citrate datasets. The plot shows the average within-cluster sum of squares (AWCSS) as a function of the number of clusters (k). The "elbow point" is the red arrow pointing to the optimal number of elbow points.*

3. Quantify the performance of the citrate-removal step In the Supporting Information, please provide Precision/Recall for predicting “citrate-positive” versus “true citrate” spectra or include an ROC curve for pure-citrate versus analyte mixtures, to demonstrate that this preprocessing does not eliminate genuine signals.

Reply: Thank you for your valuable recommendation. However, we did not consider citrate as one class for direct classification. Instead, we used it as a reference to identify and exclude citrate-affected spectra. Our original dataset, which includes data for an amino acid, its phosphorylation, and citrate, was used to provide ground truth labels for the clustering process. We noted that some spectra of the target molecules might be affected by citrate. Using k-means, we grouped the data into three clusters. The cluster containing the majority of citrate spectra was identified as contaminated and used as a reference to find other citrate-affected spectra. Consequently, **Figure S4** below and also in the revised supporting information **(page 8)** presents the relative percentages of the ground truth data and the clustered data. For the two-class clusters, the

extracted pure spectra were then used as a new ground truth label. As summarized in **Table R1**, approximately 40% of the spectra were identified as citrate affected and removed.

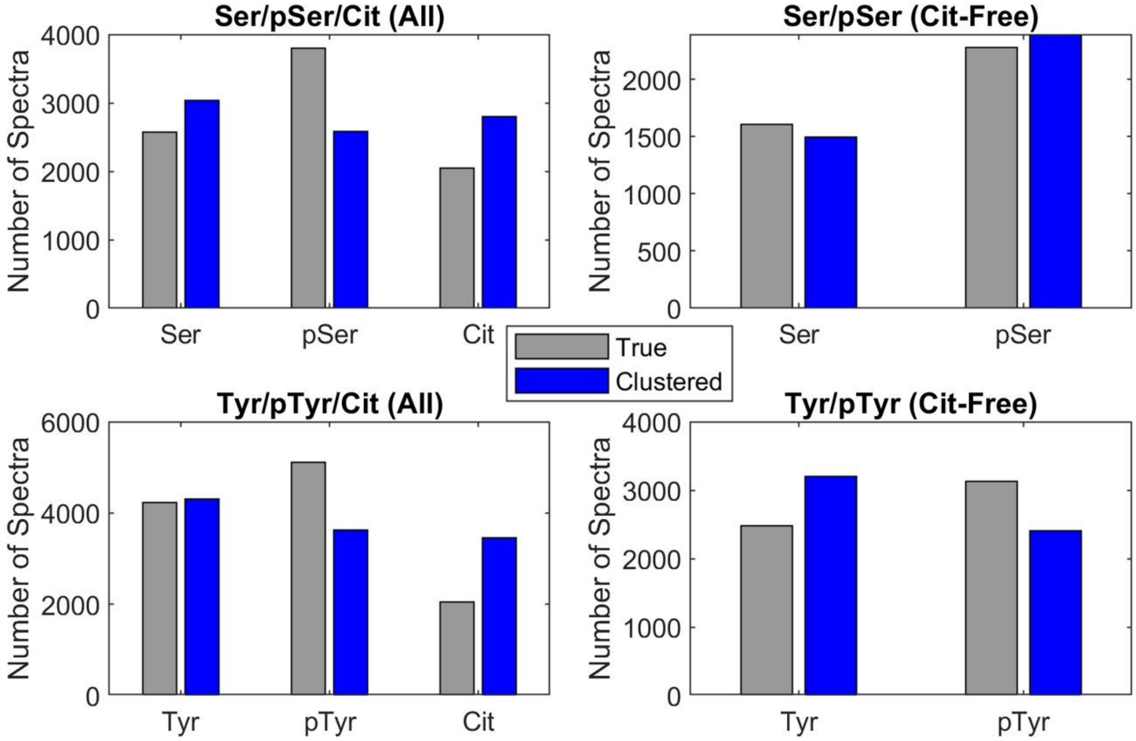

Figure S4. *The bar graph demonstrates the relative counts of SERS spectra in each cluster, with the gray bars indicating the ground truth labels and the blue bars representing the number of spectra (data points) after k-means clustering.*

Table R1. The number of spectra before and after k-means clustering; nearly 40% of spectra are citrate-affected and excluded.

Molecules	Total	Cit-free	Cit-affected	percentage of cit-affected spectra
Ser	2572	1605	967	37.6 %
pSer	3803	2281	1522	40 %
Tyr	4231	2484	1747	41.3 %

**pTyr**	**5108**	**3125**	**1983**	**38.8 %**

To demonstrate that the k-means clustering does not eliminate genuine signals, we compared the pure citrate signals with the citrate-affected ones in **Figure S5** below. Notably, these signals share a pure citrate peak at 1070 cm^{-1}, which was assigned to the v_{CO} mode of citrate.[8] We calculated the Euclidean distance between the mean spectrum of pure citrate and the mean spectra of the citrateaffected. The Euclidean distance was 2.6042 for Ser/pSer and 2.8853 for Tyr/pTyr. These low distance values, combined with the observed spectral overlap in **Figure S5**, confirm that the spectra identified as citrate-affected share a high degree of similarity with the pure citrate spectra.

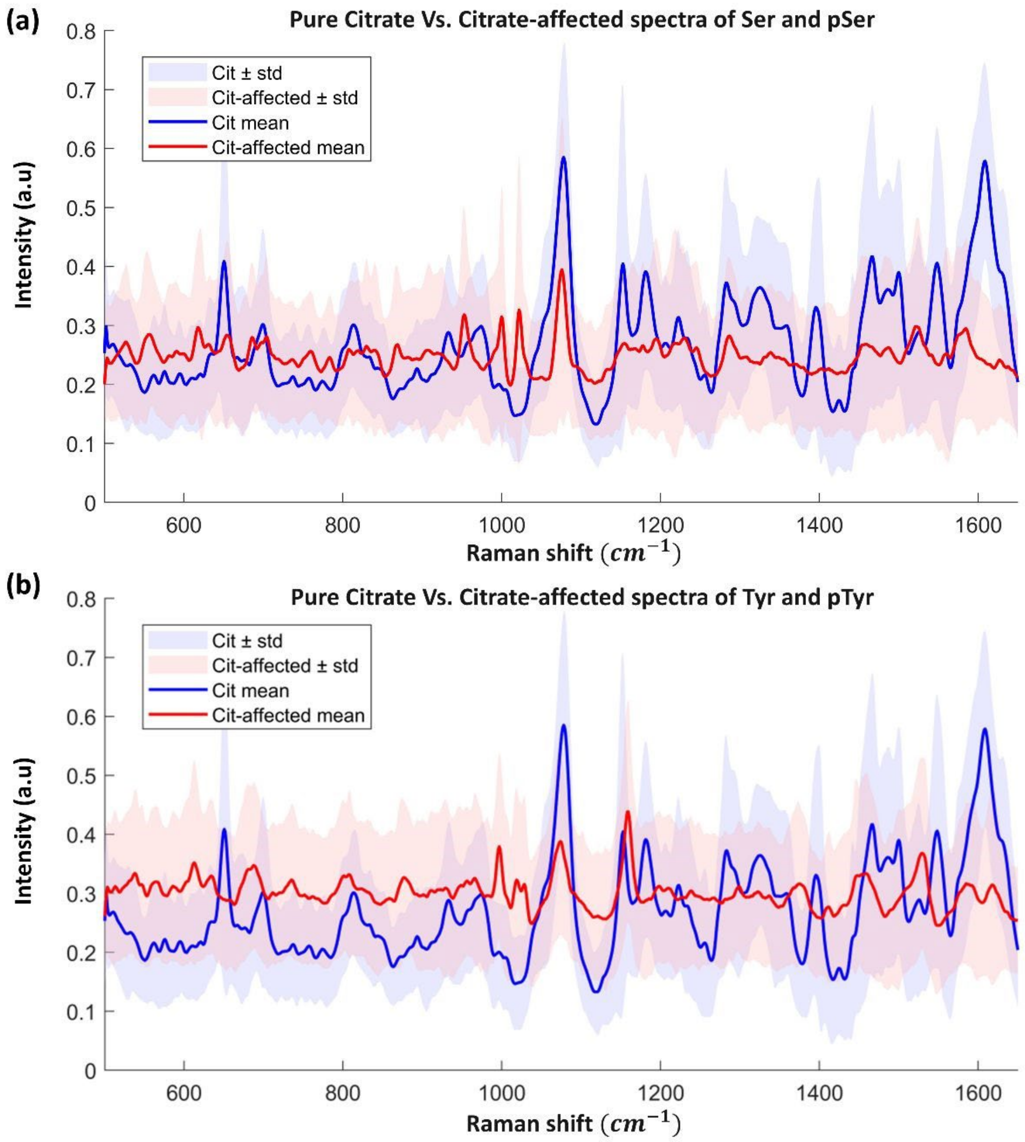

Figure S5. *Average spectra of (a) citrate and citrate-affected Ser/pSer and (b) citrate and citrateaffected Tyr/pTyr. The shaded regions represent the standard deviation at each wavenumber.*

These discussions and figures have been added to the Section: "k-means-based citrate removal" in the revised supporting information.

4. Improve figure readability The numerical labels in several figures are too small to be legible in print. Please enlarge the font sizes and, where appropriate, redraw the graphics as vector images to preserve clarity.

Reply: We sincerely appreciate your insightful and constructive comments, which have significantly improved the clarity and rigor of our manuscript. Accordingly, we have redrawn all the figures in the revised manuscript as follows:

Figure 1 **(page 4)**

Figure 2 **(page 5)**

Figure 3 **(page 8)**

Figure 4 **(page 9)**

Figure 5 **(page 10)**

5. Reference duplication and formatting issues References 16 and 25 cite the same article; DOI formats are inconsistent throughout the manuscript; several citations still use raw "http://" links instead of DOI links. Please standardize all references.

Reply: We are thankful for your careful review and the valuable perspectives you provided, which have helped us improve the quality and coherence of our manuscript. We used Mendeley Reference Manager, specifically configuring it for the *Journal of Physical Chemistry Letters*. We

have doublechecked and standardized all references, and we greatly appreciate you pointing out this area for improvement.

6. The authors have used k-means clustering and t-sne visualization to pick out the citratecontaminant spectra, but as seen from the figures (figure 3), the distribution of the citrate spectra

   and the Ser/pSer (of Tyr/pTyr) spectra were mixed together without clear boundaries, so how did the author prove the reliability of citrate removal? In addition, no matter before or after the removal of citrate-contaminated spectra, the spectra of the original amino acids and the phosphorylated amino acids showed overlapping distribution, so how significant is the improvement in classifying the two kinds of spectra by citrate removal? On the other hand, have the authors checked the citrate-contaminated spectra which was picked out by the algorithm? These data should be provided for clarity and other experimental techniques to solidate the contamination should also be used for validation.

Reply: Thank you for your valuable comments to improve our manuscript. We would like to emphasize that we employed k-means clustering to exclude citrate-affected spectra from the target molecules' spectral dataset.

We have updated Figure 3 so that we have a distinct boundary between the different clusters in the revised manuscript. To visualize the different clusters distinctly, we first implemented principal component analysis (PCA) to reduce the dimensionality of the original spectra. Each spectrum initially contained 1048 features (Raman shift intensity values), which we reduced to the 30 most significant principal components. We then applied t-distributed stochastic neighbor embedding (t-SNE), a robust non-linear dimensionality reduction technique, to visualize the spectral clustering outcomes as distinct groups in a two-dimensional space. The citrate removal process is well established and reliable as it is guided by ground truth data. K-means-based clustering has three stages: 1) clustering with the k-means algorithm; 2) identification of the contaminated cluster (i.e., the cluster containing most pure-citrate spectra); and 3) identifying citrate-affected spectra from the target molecule by iteratively searching within the contaminated cluster. The

improved performance of the k-means clustering is demonstrated by the accuracy of the one-dimensional convolutional neural network. Specifically, the model's post-evaluation performance increased after we removed the citrateaffected spectra.

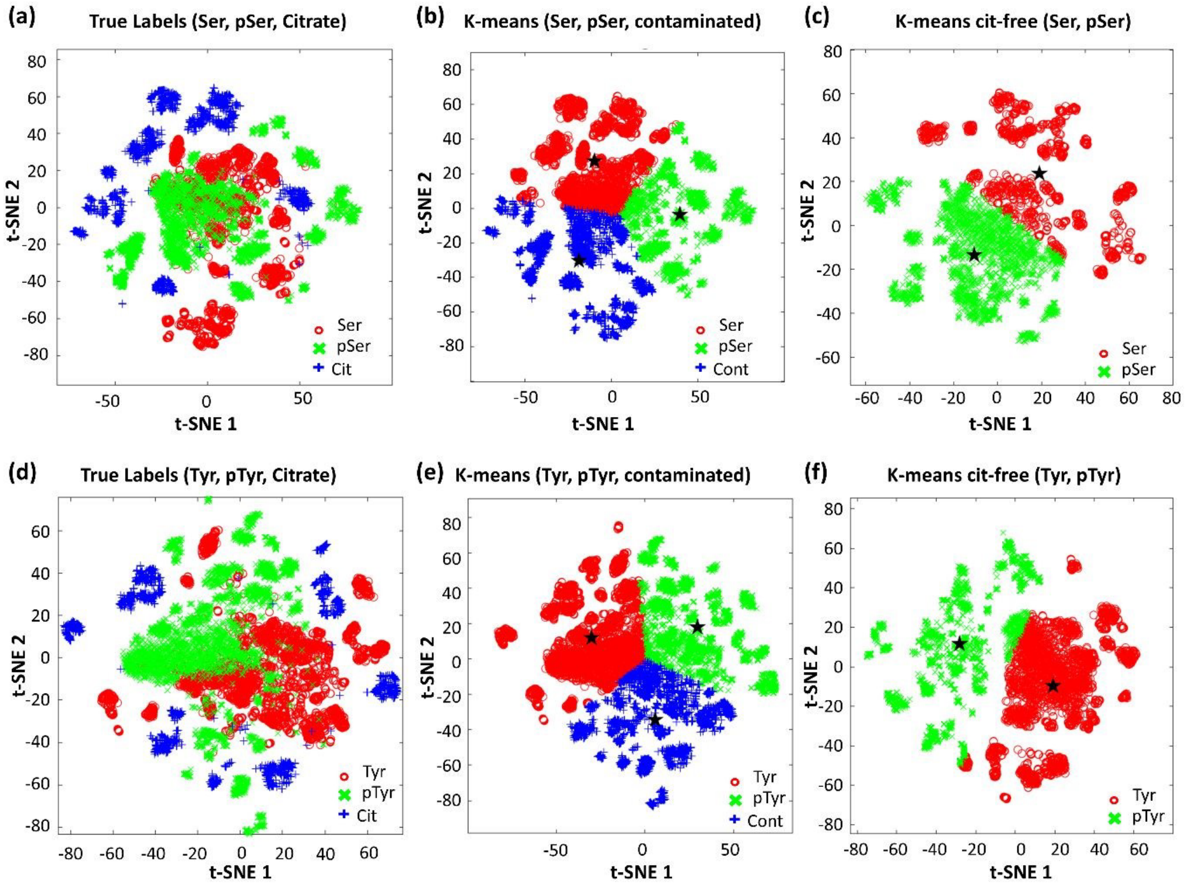

Figure 3. *Workflow of k-means-based clustering. The black star (*) indicating the centroid in each cluseter. (a) t-SNE visualization of Ser, pSer, and Cit (i.e. citrate) in two-dimensional space; (b) t-SNE visualization of k-means clustered spectra of Ser, pSer, and citrate; (c) t-SNE visualization of citratefree, k-means clustered Ser and pSer spectra; (d) t-SNE visualization of Tyr, pTyr, and citrate in twodimensional space; (e) t-SNE visualization of k-means clustered Tyr, pTyr, and citrate spectra; and (f) tSNE visualization of k-means clustered, citrate-free spectra of Tyr and pTyr.*

Table R1. The number of spectra before and after k-means clustering; nearly 40% of spectra are citrate-affected and excluded.

Molecules	Total	Cit-free	Cit-affected	percentage of cit-affected spectra
Ser	2572	1605	967	37.6 %
pSer	3803	2281	1522	40 %
Tyr	4231	2484	1747	41.3 %
pTyr	5108	3125	1983	38.8 %

We got about 40 % of the spectra as citrate-contaminated in **Table R1.** To demonstrate that the kmeans clustering does not eliminate genuine signals, we compared the pure citrate signals with the citrate-affected ones in **Figure S5** below. Notably, these signals share a pure citrate peak at 1070 cm^{1}, which was assigned to the v_{CO} mode of citrate.[8] We calculated the Euclidean distance between the mean spectrum of pure citrate and the mean spectra of the citrate-affected. The Euclidean distance was 2.6042 for Ser/pSer and 2.8853 for Tyr/pTyr. These low distance values, combined with the observed spectral overlap in **Figure S5**, confirm that the spectra identified as citrate-affected share a high degree of similarity with the pure citrate spectra.

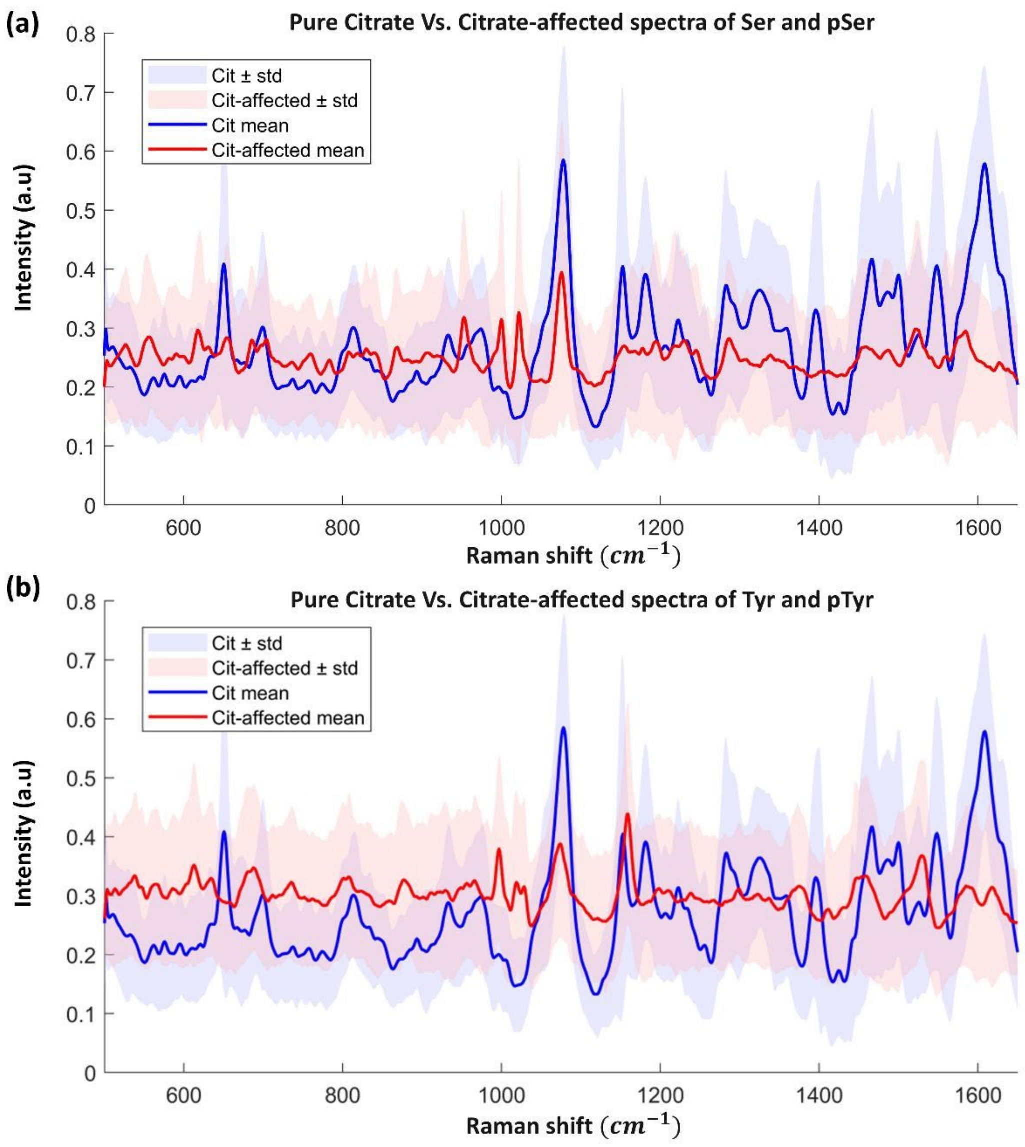

Figure S5. *Average spectra of (a) citrate and citrate-affected Ser/pSer and (b) citrate and citrateaffected Tyr/pTyr. The shaded regions represent the standard deviation at each wavenumber.*

These discussions and figures have been added to the Section: “k-means-based citrate removal” in the revised supporting information, while those of updated Figure 3 have been added to the revised manuscript.

7. Please cite the recent very relevant review article (ACS Appl. Mater. Interfaces 2025, 17, 11, 16287–16379) and the research article (Digital colloid-enhanced Raman spectroscopy by singlemolecule counting, Nature, 2024, 628, 771). For the pSer and pTyr, can the peak occurrence frequency also be digitalized, which will be easily counted? The study represents a clear advance in single-molecule phosphosite detection, with a complete and convincing experimental– computational workflow. The issues raised above are chiefly clarifications or additions that do not alter the main conclusions. I therefore recommend acceptance after Minor Revision.

Reply: Thank you for your positive decision and for the helpful suggestion to include more recent publications in the field of single-molecule detection. We have now cited the papers you mentioned as reference No. 12 and 13 in the revised manuscript. Regarding the peak occurrence frequency, yes it can be digitalized at a specific wavenumber for further quantification of the amino acids and their phosphorylation in the future. In current work, we normalized this frequency by dividing the count for each peak by the total number of spectra for spectral feature study only for the single-molecule phosphorylation.

Additional Questions:

Urgency: High

Significance: Top 10%

Novelty: Top 10%

Scholarly Presentation: High

Is the paper likely to interest a substantial number of physical chemists, not just specialists working in the authors' area of research? Yes

Reviewer: 2

Recommendation: This paper is publishable subject to minor revisions noted. Further review is not needed.

Comments:

General comments: The authors developed a plasmonic particle-in-pore sensor to obtain singlemolecule SERS spectra of phosphorylation at Serine and Tyrosine, and the result spectral data was analyzed through deep learning method. The major advance of this manuscript is that discriminating the phosphorylation in Serine and Tyrosine with over 95% and 97% accuracies, respectively. The author should pay attention that efforts in single-molecule PTM identification have recently emerged, and the novelty claim in the paper should be cautiously positioned. This manuscript can be published on The Journal of Physical Chemistry Letters after addressing the following concerns:

1. why does the Raman peak of spectra in Figure 2a show obvious shift especially around 900 and 1200 cm-1?

Reply: We are thankful for your careful review and the valuable perspectives you provided to improve our manuscript.

Spectral fluctuation is a natural characteristic of single-molecule SERS. The Raman peak shifts observed around 900 cm^{-1} and 1200 cm^{-1} are attributed to the asymmetric stretching of P-O and the bending of C-O-H, respectively.[1,2]

In our particle-in-pore sensor, gold nanoparticles are trapped in close proximity to the gold sidewall of the nanopore. The adsorption and desorption of gold nanoparticles in the nanopore create fluctuations in the SERS spectra of the target molecules. Additionally, when the molecules diffuse on the gold nanoparticle, they may access the hot spot in different conformations, which also results in fluctuations in the SERS spectra. The molecular orientation on the plasmonic hot spot also creates differences in the relative peak intensities between the two spectra as shown in **Figure R4**.[3,4] In another case, peak shifts may occur likely due to charge transfer interactions between target molecule and the gold surface in different orientations.[5]

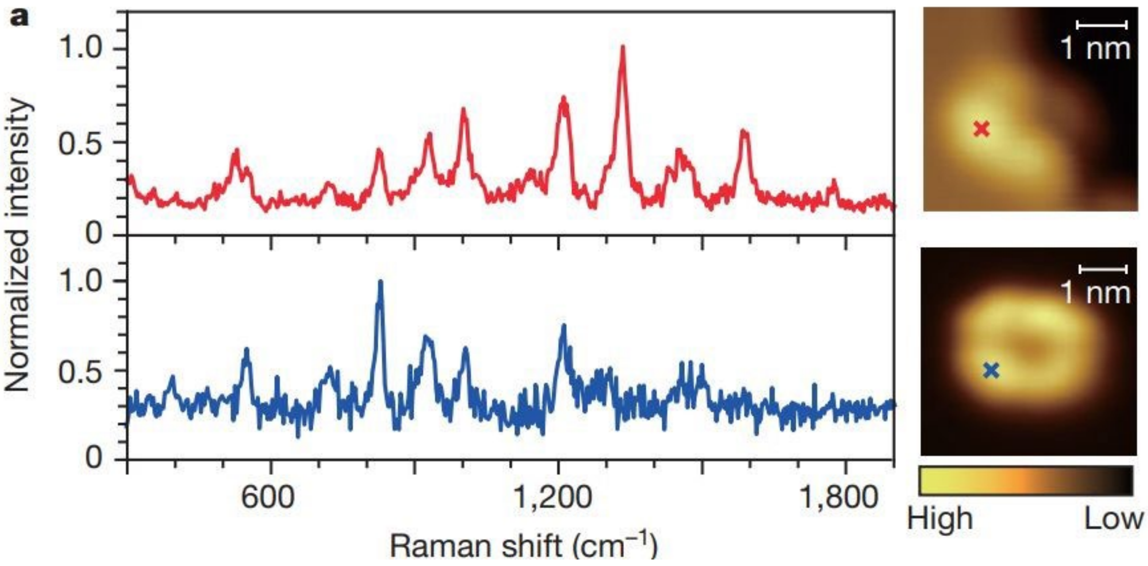

Figure R4 *Single-molecule TERS spectra and their dependency on molecular orientations. a, Singlemolecule TERS spectra (100 mV, 1 nA, 3 s) for an isolated H2TBPP molecule adsorbed on the terrace (bottom, blue) or at the step edge (top, red) of Ag (111). Both spectra were acquired on the molecular lobes marked with crosses in the STM images on the right (subtracted from the broad continuum for clarity).*[3]

The above discussion and revision have been added to address the fluctuation nature of SM-SERS in the revised manuscript (**page 4-5**). Furthermore, the peak assignments at approximately 900 cm^{-1} and 1200 cm^{-1} are based on **Table 1** on **page (11–12)** of the revised manuscript.

"Unlike multi-molecule SERS spectra where spectral features are averaged over many molecules, SMSERS spectra exhibit significant temporal fluctuations in peak position, intensity, and bandwidth. This variablility is due to the probabilistic nature of molecular adsorption within the hot spots. Additionally, peak shifts can occur due to charge transfer interactions between the target molecule and the gold surface in different orientations"

2. In Figure 4d, the highlighted spectral regions from Grad-CAM do not always correspond to clear Raman peaks or chemically meaningful vibrational modes. The authors should discuss the possible implications of this.

Reply: we thank you for your careful review and insightful questions. Grad-CAM highlights the key spectral features in one dimensional convolutional neural network that has high feature weight to distinguish between spectra of an amino acid and its phosphorylated one mathematically. In a classification problem, the model focuses on features that maximize the discrimination between the classes based on their mathematical difference. Therefore, the spectral dips may contribute a significate feature weight in identifying the two classes, and these regions cannot be assigned to a specific vibrational mode. In other cases, the Grad-CAM values may correspond to distinct spectral peaks related to the molecular structure, which we can then assign to a known vibrational mode.

We updated the discussion on the revised manuscript (**page 12)** as shown below.

*"In fact, the distinct differences in the high Grad-CAM features could correspond to either inherent molecular vibrations of the two molecules or just data differences without corresponding vibrational modes. A good example of the latter is the high pTyr Grad-CAM feature at around 1027 cm^{-1} that does not correspond to a high SM-SERS peak occurrence frequency in **Figure 5d**. Therefore, only those Grad-CAM regions overlapping with high SMSERS peak occurrence frequency were confirmed to represent molecular structural differences and can be assigned to a certain vibrational mode. Due to the lack of a comprehensive spectral database for tyrosine and serine phosphorylation, some frequently occurring peaks may not be assigned."*

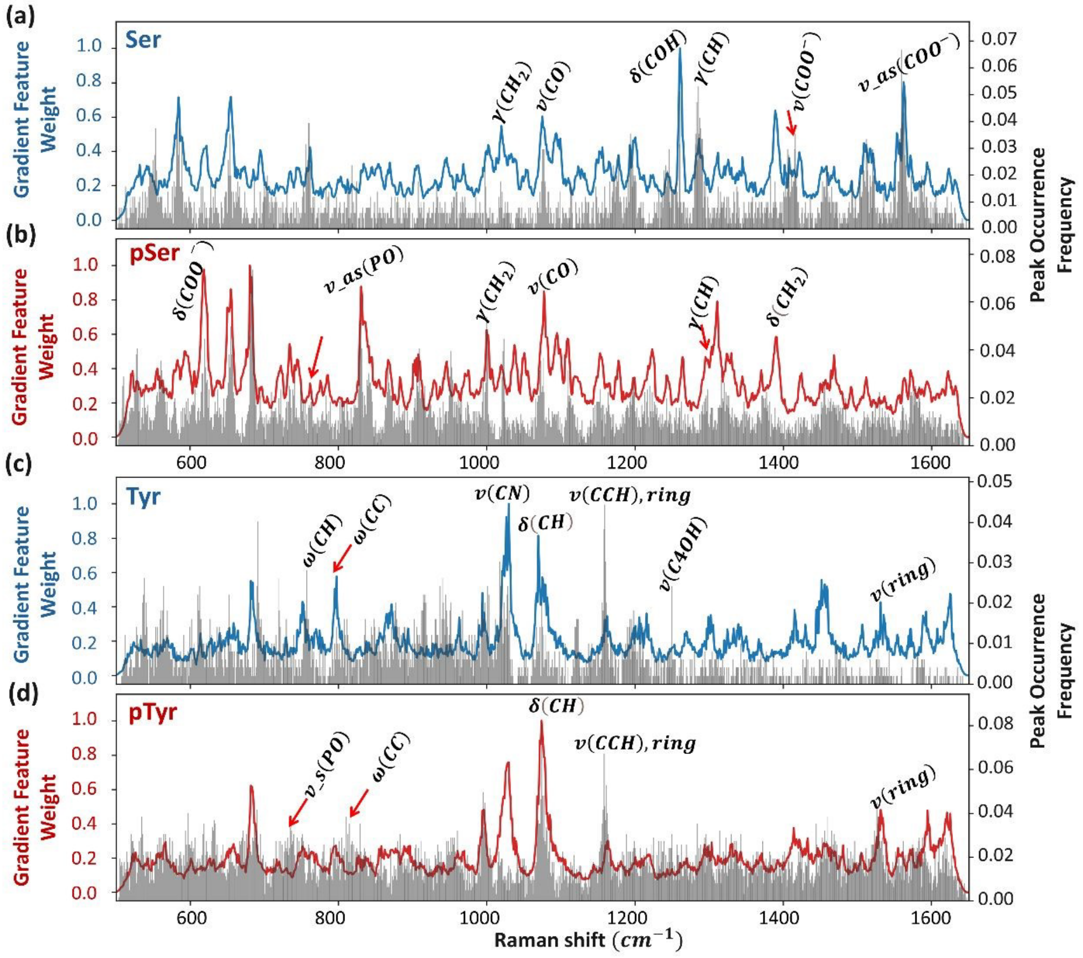

__Figure 5.__ Normalized Grad-CAM feature weights extracted by the 1D-CNN model with histogram of peak occurrence frequency of entire citrate-free dataset. The blue curve in (a) represents the 1D GradCAM feature weights for Ser, while the red curve in (b) shows those for pSer. Similarly, the blue curve in (c) corresponds to Tyr, and the red curve in (d) to pTyr. Gray spikes in each panel indicate the peak occurrence frequencies of the corresponding molecules.

3. While the k-means filtering step effectively reduces citrate background, it would be helpful to explicitly describe the rationale for selecting 4 clusters. Did the authors test other cluster numbers or validate the citrate-related cluster using known references?

Reply: Thank you for your careful review and valuable feedback. We have revised the manuscript to clarify our methodology for the k-means filtering step.

Based on our prior knowledge, the datasets were known to contain three clusters: the amino acid, its phosphorylated form, and citrate. We therefore anticipated three distinct spectral clusters. We validated it using the elbow method, which quantitatively supported three as the optimal number of clusters as shown in **Figure S3** below. The cluster containing contaminated spectra was identified by mode function in MATLAB based on the reference citrate spectral features and then the citrateaffected spectra are excluded by searching them iteratively in the contaminated cluster.

Figure S3 as the rationale for choosing the 3 clusters for k-means clustering has been added in to the revised supporting information **on page 6**.

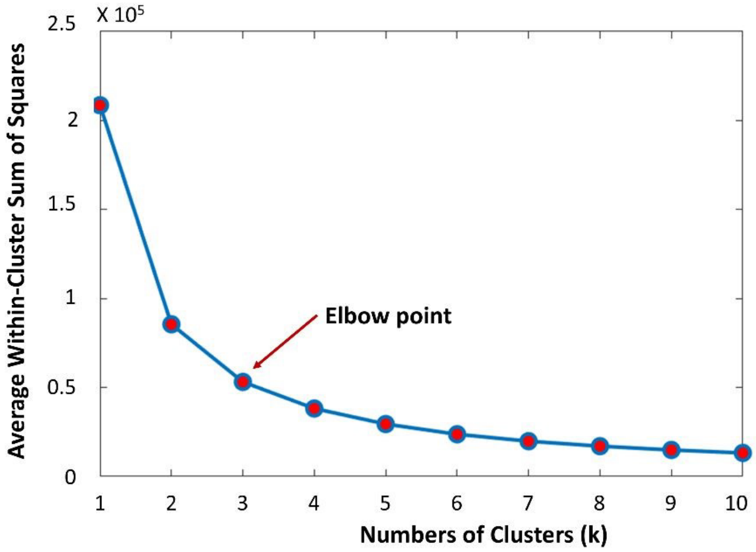

***Figure S3**. Elbow Method for determining the optimal number of clusters (k) on Ser, pSer, and Citrate datasets. The plot shows the average within-cluster sum of squares (AWCSS) as a function of number of clusters (k). The "elbow point" is the red arrow pointing the optimal number of elbow points.*

4. What are the specific aspects of its chemical specificity of SM-SERS compared with multi-molecule detection? For the proof of chemical selectivity, there are some literatures for reference, Nat. Commun. 2024, 15: 5855.

Reply: We sincerely appreciate your comments and concern about the chemical specificity of SM-SERS and multimolecular SERS. Our method has the same chemical specificity as common

nanoparticlebased multimolecular SERS systems. In our particle-in-pore sensor, the analyte molecules were physically adsorbed on the gold nanoparticle surface by 2-day incubation before the nanoparticle was trapped in the gold nanopore for single-molecule SERS detection. We also appreciate your recommendation of the literature to prove the single molecule sensitivity and selectivity, which is now cited as reference No. 20 in the manuscript.

5. The approach works well for pSer/pTyr classification, but how general is the model across different peptide sequences or environments? A short discussion on model transferability or limitations would add depth.

Reply: Thank you so much for dedicating your time to reviewing our manuscript and providing us with valuable and constructive feedback. In this work, we didn't validate the model based on the peptide dataset. Peptide and protein have long sequences and their phosphorylation's have many amino acids in common, the PTM site might not necessarily be located in the hot spot for SERS detection. This leads to heavy overlaps in the spectra of the peptide and its PTMs. Therefore, we may need a deeper and more complex tuning of the model architecture to differentiate peptide spectra effectively.

We added the above discussion on model transferability for peptide data to the revised manuscript **(page 15)** as shown below.

"The current model can be further customized for peptide and protein sequence analysis. Hence peptide has a long sequence and their phosphorylation's have many amino acids in common, the PTM site might not necessarily be located in the hot spot for SERS detection. This leads to heavy overlaps in the spectra of the peptide and its PTMs. Therefore, we may need a deeper and more complex tuning of the model architecture to differentiate peptide spectra effectively."

6. Others, Figure 2d (confusion matrix) should specify which label corresponds to which class (pSer, pTyr) more clearly; Figure 3c-d (t-SNE plots) could benefit from color bars or consistent legends to enhance readability; please clarify how "single-molecule" was determined; Please

ensure that training data and model code (or a minimal implementation) are made publicly accessible;

Reply: Thank you so much for reviewing our manuscript and providing us with valuable and constructive feedback. We have updated all the figures as presented as reply for questions number 3 of reviewer 1. The single molecule sensitivity of particle-in-pore sensor has been determined by the bi-analyte SERS (BIASERS) technique in our previous papers.[6] The model has been uploaded in our Github (https://github.com/MulusewWondie/Single-molecule-Phosphorylation-Identification-) for free access, while the training data can be obtained on request.

Additional Questions: Urgency:

High

Significance: Top 10%

Novelty: High

Scholarly Presentation: Top 10%

Is the paper likely to interest a substantial number of physical chemists, not just specialists working in the authors' area of research? Yes

Reviewer: 3

Recommendation: This paper may be publishable, but major revision is needed; I would like to be invited to review any future revision.

Comments:

The manuscript presents a novel approach single-molecule SERS combined with deep learning for detecting phosphorylation in serine and tyrosine. While the integration of plasmonic nanopore sensors and machine learning is a very effective approach and potentially impactful, the

manuscript suffers from several critical issues in terms of scientific rigor, clarity, and validation. In its current form, the manuscript is not suitable for publication.

1. The authors claim this is the first demonstration of single molecular SERS (SM-SERS) detection of pSer and pTyr. However, similar approaches using SERS and machine learning for PTM detection have been reported. The manuscript fails to clearly differentiate this work from prior studies (e.g., Zhao et al., Nano Lett. 2025). And also, deep learning has been widely applied in SERS analyses, and the authors should justify why current approach was chosen over other architectures.

Reply: Thank you so much for your time to reviewing our manuscript and providing us with valuable and constructive feedback. Despite of using the same particle-in-pore sensors, the difference between current study and the prior study (Zhao et al., Nano Lett. 2025) lies in the removal methods of interference of the citrate signals on the single-molecule SERS spectra. The prior study (Zhao et al., Nano Lett. 2025) must replace the citrate with 48-hour incubation with monolayer of the analyte (Proline or Hydroxyproline) on the nanoparticle surface, while current study uses k-means clustering to exclude the citrate-contaminated spectra from the submonolayer of analytes (amino acids and their phosphorylation) with 1.23% nanoparticle surface coverage. While the PTMs are different (phosphorylation VS hydroxylation), current work can have much lower particle surface coverage, i.e.
1.23%, than the 100% coverage by analyte monolayer in the prior study (Zhao et al., Nano Lett. 2025).

We added the quoted text below to the revised manuscript **(page 5-6).**

"In our previous paper, we identified hydroxylation at the single molecule level, whereas this work focuses on the phosphorylation of serine and tyrosine. The citrate interference is a bottleneck for single molecule data analysis. Previously, we experimentally mitigated this issue by substituting citrate with an analyte monolayer. However, this approach has two key limitations. First, it does not completely eliminate citrates, leading to residual interference as citrate could still access the hot spot. Second, the requirement of a substantial number of analyte molecules to form a complete monolayer on the nanoparticle limits further improvements in detection sensitivity.

To address these limitations, we engineered the platform such that the analyte molecule occupied only

1.23% of the particle surface. The remaining surface was covered by citrates, which generated SMSERS noise when excited by the hot spot. To preclude citrate influence, we implemented a k-meansbased clustering algorithm to exclude citrate-contaminated spectra from our SM-SERS datasets. The k-means-based clustering has three stages: 1) clustering with the k-means algorithm; 2) identification of the contaminated cluster (i.e., the cluster containing most of pure-citrate spectra); and 3) identifying citrate-affected spectra from the target molecule by iteratively searching within the contaminated cluster."

2. The reported analyte surface coverage of 1.23% is extremely low. The authors should provide quantitative evidence (e.g., surface density measurements or other control experiments) to support the claim that meaningful SM-SERS signals can be reliably obtained under such sparse conditions. In addition, the reproducibility of the SM-SERS measurements is not addressed. How many independent experiments were conducted?

Reply: Thank you so much for reviewing our manuscript and providing us with valuable and constructive feedback.

While the 1.23% surface coverage is difficult to be measured precisely and not the focus of current work, such surface coverage was supported by our previous publications using a published biomolecular database.[7] Accordingly, the experiment was conducted using a gold nanoparticle with a 50 nm diameter. Thus, surface area of the gold nanoparticle will be $A = 4\pi r^2 = 7850\ nm^2$. For the Serine with its maximum accessible surface area of 1.43 nm^2, only 68 Serine are adsorbed on the particle surface on average. The relative coverage of Serine on the gold nanoparticle can be calculated

as; $Coverage = \frac{total\ surface\ area\ of\ Serine}{Surface\ area\ of\ the\ Au} = \frac{1.43 * 68}{7850} = 0.0123$ that means only 1.23% of gold maybe

covered Serine. The detailed calculations of Tyr, pTyr and pSer have been included in the supporting information **Table S1(page 2).** With this indirect evidence, we loosened our claim of

1.23% surface coverage by replacing it with “submonolayer analyte coverage of particle surface” in the Abstract.

The SM-SERS measurement in our particle-in-pore platform are quite reproducible, as we collected more than 20,000 SM-SERS spectra for each molecule (Citrate, Ser, Tyr, pSer, pTyr). We used 10 nanopores for each type of molecules. For an independent measurement, a new particle would be trapped into the nanopore to produce the SM-SERS spectra time series. We had considered 13 independent measurements for citrate, 11 for Tyrosine, 23 for phosphorylated Tyrosine, 13 for Serine, and 15 measurements for phosphorylated serine. Each independent measurement contains 2000 spectra, while some yield a better signal than others depending on the molecular structures. These descriptions of reproducible SM-SERS measurement have been added to the revised manuscript (page 5).

3. The use of k-means clustering for citrate signal removal is inadequately justified. How was the number of clusters selected? Were alternative clustering methods considered? The CNN architecture is only described in the Supporting Information. The main text should include a concise but complete description of the model, including layer types, activation functions, and regularization strategies.

Reply: Thank you so much for providing valuable and constructive feedback.

From our prior knowledge on the data, which included an amino acid, its phosphorylation, and citrate. We set the number of clusters to be three, we also validated it with the elbow method in **Figure S3** below and also in the revised supporting information **(page 6)**. We did not consider other clustering algorithms; we established a k-means-based clustering technique to exclude citrate-affected spectra. The k-means-based clustering has three stages: 1) clustering with the k-means algorithm, 2) identification of the contaminated cluster (i.e., the cluster containing the most pure-citrate spectra), and 3) an iterative search for the belonging of spectra from the target molecule within the contaminated cluster.

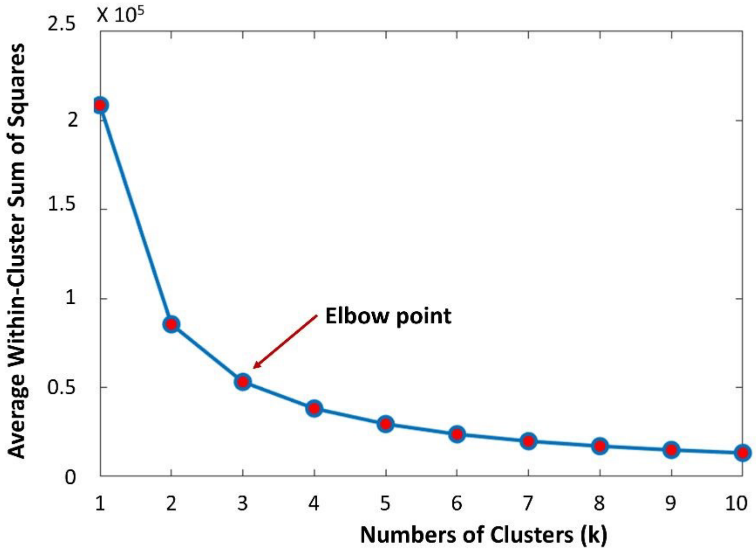

***Figure S3**. Elbow Method for determining the optimal number of clusters (k) on Ser, pSer, and Citrate datasets. The plot shows the average within-cluster sum of squares (AWCSS) as a function of number of clusters (k). The "elbow point” is the red arrow pointing the optimal number of elbow points.*

Consequently, **Figure S4** below and also in the revised supporting information **(page 8)** presents the relative percentages of the ground truth data and the clustered data. For the two-class clusters, the extracted pure spectra were then used as a new ground truth label. As summarized in **Table R1**, approximately 40% of the spectra were identified as citrate affected and removed.

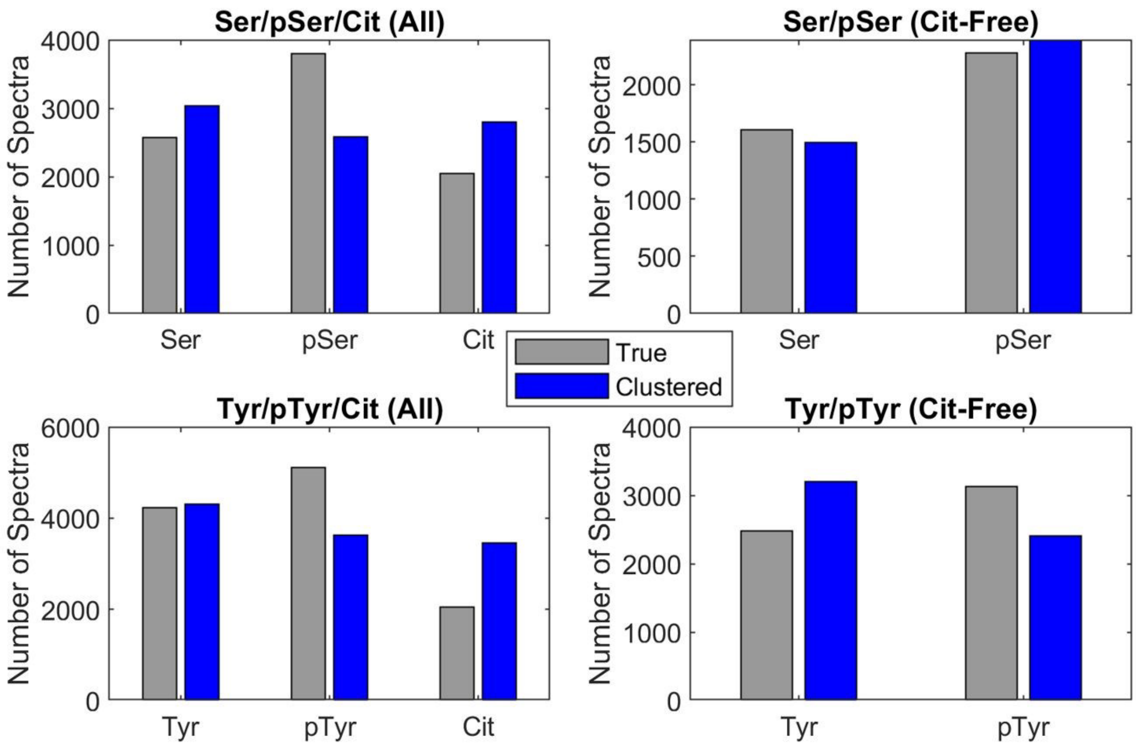

Figure S4. *The bar graph demonstrates the relative counts of SERS spectra in each cluster, with the gray bars indicating the ground truth labels and the blue bars representing the number of spectra (data points) after k-means clustering.*

Table R1. The number of spectra before and after k-means clustering; nearly 40% of spectra are citrate-affected and excluded.

**Molecules**	**Total**	**Cit-free**	**Cit-affected**	**percentage of cit-affected spectra**
**Ser**	**2572**	**1605**	**967**	**37.6 %**
**pSer**	**3803**	**2281**	**1522**	**40 %**
**Tyr**	**4231**	**2484**	**1747**	**41.3 %**
**pTyr**	**5108**	**3125**	**1983**	**38.8 %**

To demonstrate that effectiveness of the k-means clustering, we compared the pure citrate signals with the citrate-affected ones in **Figure S5** below. Notably, these signals share a pure citrate peak at 1070 cm^{-1}, which was assigned to the ν_{CO} mode of citrate.[8] We calculated the Euclidean distance between the mean spectrum of pure citrate and the mean spectra of the citrate-affected. The Euclidean distance was 2.6042 for Ser/pSer and 2.8853 for Tyr/pTyr. These low distance values, combined with the observed spectral overlap in **Figure S5**, confirm that the spectra identified as citrate-affected share a high degree of similarity with the pure citrate spectra.

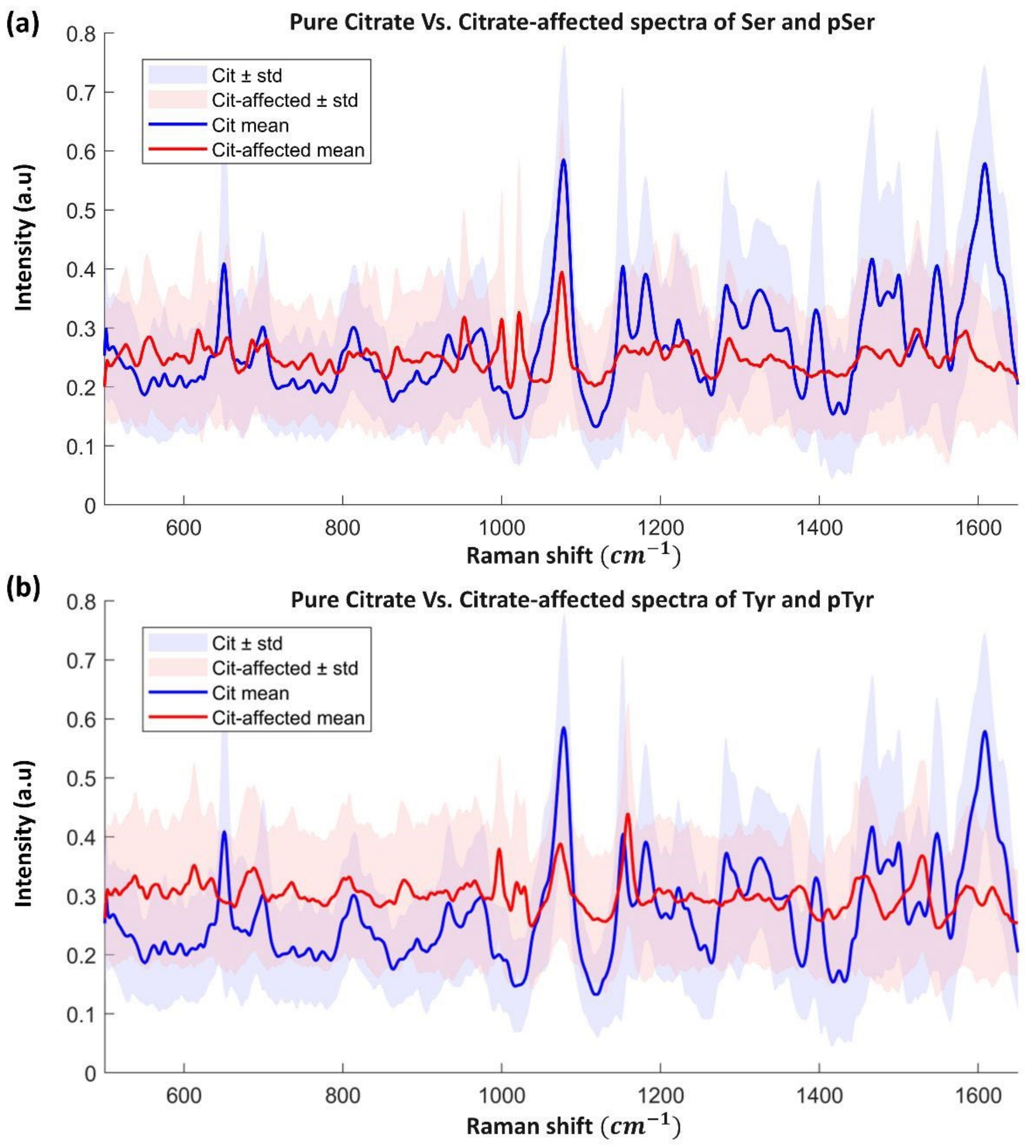

Figure S5. *Average spectra of (a) citrate and citrate-affected Ser/pSer and (b) citrate and citrateaffected Tyr/pTyr. The shaded regions represent the standard deviation at each wavenumber.*

These discussions and figures have been added to the Section: "k-means-based citrate removal" in the revised supporting information.

For CNN architect, we have added a short description of the CNN architect to the revised manuscript **(page 7) as shown below** according to your suggestions.

"Our 1D-CNN model consists of an input, three convolutional blocks, a flattening layer, two fully connected blocks (dense blocks), and an output layer. Each convolutional block consists of two convolutional layers, two batch normalization (BN) layers, the max-pooling layer, and dropout layers. We started a convolutional layer with 16 filters and proceeded to a convolutional layer having 64 filters by doubling each time. Batch normalization accelerates convergence, while max-pooling reduces spatial dimensions and dropout prevents overfitting. The flattening layer is used to transform multiple feature maps produced by the convolutional layers into 1D vector. The rectifier linear unit (ReLU) activation function introduces non-linearity to the model and mitigates the vanishing gradient and l2 kernel regularization is used. The fully connected (Dense layer) basically do the final decision after the convolutional and pooling layers extract features, which should learn the global patterns in their input feature spaces to classify them. Finally, the Sigmoid activation function in the output layer is used to convert raw score outputs into a probability distribution over the binary classes."

4. The Grad-CAM analysis is interesting but lacks rigorous interpretation. Several highlighted regions do not correspond to known vibrational modes, raising concerns about model overfitting or spurious correlations. The authors claim high accuracy (>95%) but do not report confidence intervals or statistical significance. Given the relatively small dataset (especially

after citrate removal). Here are the minor comments: The authors should clarify whether the same CNN model was used for both Ser/pSer and Tyr/pTyr classification tasks. The ROC curves in Figure 4 are not clearly labeled. AUC values should be reported. The GitHub link provided is incomplete and should be verified.

Reply: Thank you so much for dedicating your time to reviewing our manuscript and providing us with valuable and constructive feedback to improve our work.

For the highlighted regions in the Grad-CAM spectra that were not assigned, we assume the following reasons. First, Grad-CAM's visualization is dependent on how the model makes its classification mathematically. This means the model may focus on features with obvious mathematical difference, such as dips or subtle spectral shifts. If those features are crucial for maximizing discrimination between classes.[8–10] In such cases, these features may not be easily highlighted or assigned to a specific spectral peak. Second, there is a lack of a comprehensive spectral database for single molecule sensing of Tyrosine, Serine, and their phosphorylated forms. Most existing databases are derived from multimolecular (ensemble) measurements, which show a limited number of averaged peaks. In contrast, our single-molecule sensors are extremely sensitive to a molecule's conformational changes or its orientation relative to the hot spot, which results in spectra with multiple peaks. Therefore, a direct comparison to ensemble databases is not feasible for assigning all observed peaks.

The above discussion of Grad-CAM analysis has been added to the revised manuscript (**page 10**) as shown below:

*"In fact, the distinct differences in the high Grad-CAM features could correspond to either inherent molecular vibrations of the two molecules or just data differences without corresponding vibrational modes. A good example of the latter is the high pTyr Grad-CAM feature at around 1027 cm^{-1} that does not correspond to a high SM-SERS peak occurrence frequency in **Figure 5d**. Therefore, only those GradCAM regions overlapping with high SM-SERS peak occurrence frequency were confirmed to represent molecular structural differences and can be assigned to a certain vibrational mode. Due to the lack of a comprehensive spectral database for tyrosine and serine phosphorylation, some frequently occurring peaks may not be assigned."*

We used the same CNN architecture to discriminate between Tyrosine and phosphorylated Tyrosine as we did for Serine and phosphorylated Serine.

We endorse your comments on the ROC labels and have re-labeled them as requested in the Figure 4 below. We also report AUC, Precision, and Recall values in the revised supporting information **(page 12)** as shown on **Table S4 as shown below**. We have also provided the scripts code used for the analysis in the updated and validated GitHub link (https://github.com/MulusewWondie/Singlemolecule-Phosphorylation-Identification-).

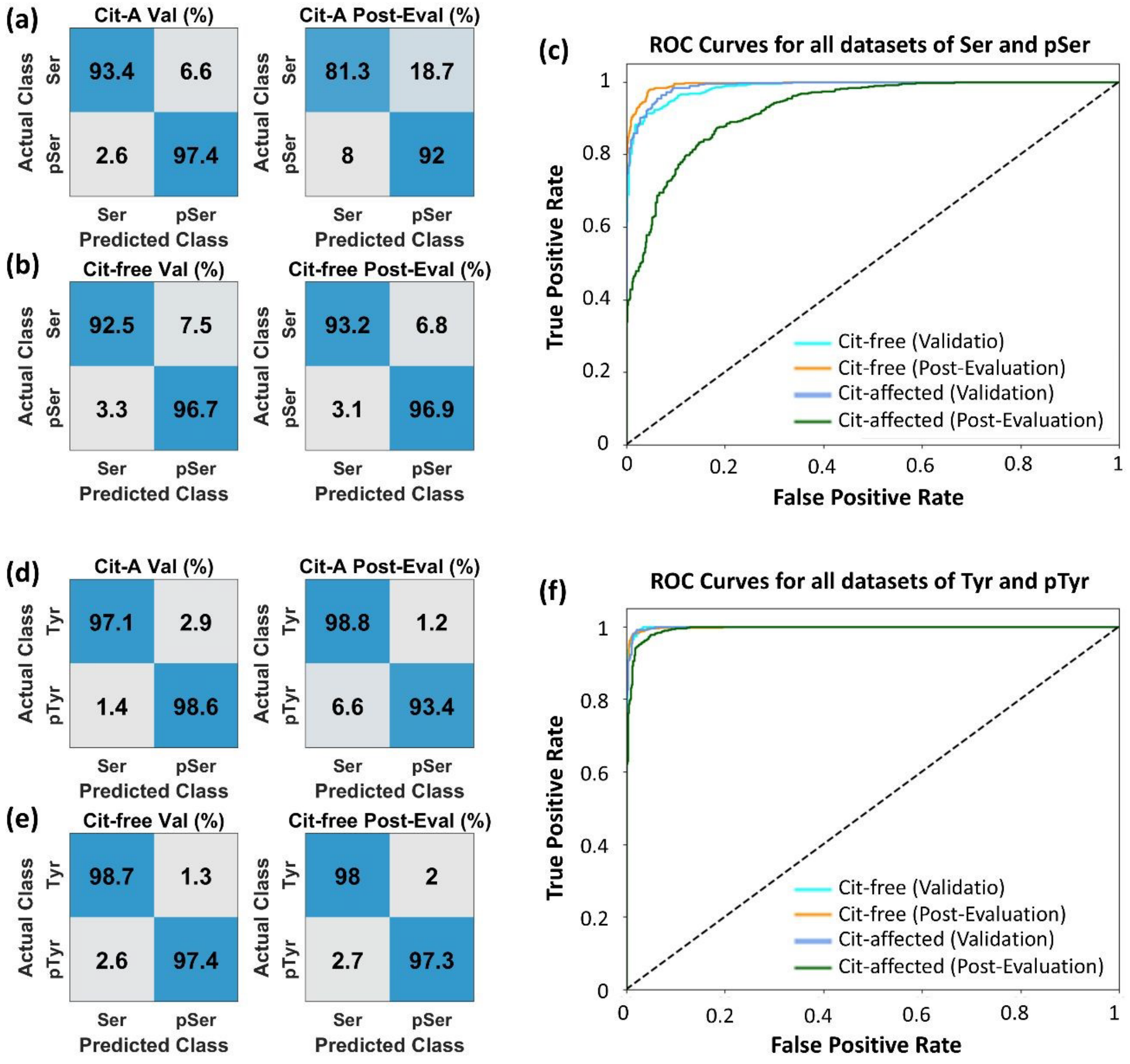

Figure 4*. The performance metrics of the 1D-CNN on the identification of Ser from pSer and Tyr from pTyr. The confusion matrice show the classfication accuracies at validation (Val) and post-*

evaluation (Post-Eval) stages of (a,d) citrate-affected spectra (Cit-A) and (b,e) citrate-free spectra(Cit-free), respectively. (c,f) The corresponding ROC curve on *citrate-affected and citrate-free spectra on the validation and post-evaluation sets.*

Table S4: Performance of the model on the training and post-evaluation sets for both citrate-free and citrate-affected spectra. Added in the supporting information (**page 12**)

Dataset (Ser Vs. pSer)	AUC (%)	Precision (%)	Recall (%)
Cit-free Training	99.08	97.89	97.32
Cit-free post-evaluation	97.15	99.09	94.86
Cit-affected Training	98.89	94.98	97.59
Cit-affected post-evaluation	86.24	95.04	91.01
Dataset (Tyr Vs. pTyr)	AUC (%)	Precision (%)	Recall (%)
Cit-free Training	99.74	98.19	97.52
Cit-free post-evaluation	98.71	98.71	98.08
Cit-affected Training	99.48	96.98	96.90
Cit-affected post-evaluation	92.32	97.76	99.38

Urgency: Moderate

Significance: Moderate

Novelty: Moderate

Scholarly Presentation: Moderate

Is the paper likely to interest a substantial number of physical chemists, not just specialists working in the authors' area of research?: Yes

References

(1) Andrushchenko, V.; Benda, L.; Páv, O.; Dračínský, M.; Bouř, P. Vibrational Properties of the Phosphate Group Investigated by Molecular Dynamics and Density Functional Theory. *J Phys Chem B* **2015**, *119* (33), 10682–10692. https://doi.org/10.1021/acs.jpcb.5b05124.

(2) Jarmelo, S.; Reva, I.; Carey, P. R.; Fausto, R. Infrared and Raman Spectroscopic Characterization of the Hydrogen-Bonding Network in l-Serine Crystal. *Vib Spectrosc* **2007**, *43* (2), 395–404. https://doi.org/10.1016/j.vibspec.2006.04.025.

(3) Zhang, R.; Zhang, Y.; Dong, Z. C.; Jiang, S.; Zhang, C.; Chen, L. G.; Zhang, L.; Liao, Y.; Aizpurua, J.; Luo, Y.; Yang, J. L.; Hou, J. G. Chemical Mapping of a Single Molecule by Plasmon-Enhanced Raman Scattering. *Nature* **2013**, *498* (7452), 82–86. https://doi.org/10.1038/nature12151.

(4) Pieczonka, N. P. W.; Aroca, R. F. Single Molecule Analysis by Surfaced-Enhanced Raman Scattering. *Chem Soc Rev* **2008**, *37* (5), 946. https://doi.org/10.1039/b709739p.

(5) Zou, Y.; Jin, H.; Ma, Q.; Zheng, Z.; Weng, S.; Kolataj, K.; Acuna, G.; Bald, I.; Garoli, D. Advances and Applications of Dynamic Surface-Enhanced Raman Spectroscopy (SERS) for Single Molecule Studies. *Nanoscale* **2025**, *17* (7), 3656–3670. https://doi.org/10.1039/D4NR04239E.

(6) Huang, J.-A.; Mousavi, M. Z.; Zhao, Y.; Hubarevich, A.; Omeis, F.; Giovannini, G.; Schütte, M.; Garoli, D.; De Angelis, F. SERS Discrimination of Single DNA Bases in Single Oligonucleotides by Electro-Plasmonic Trapping. *Nat Commun* **2019**, *10* (1), 5321. https://doi.org/10.1038/s41467-019-13242-x.

(7) Huang, J.; Mousavi, M. Z.; Giovannini, G.; Zhao, Y.; Hubarevich, A.; Soler, M. A.; Rocchia, W.; Garoli, D.; De Angelis, F. Multiplexed Discrimination of Single Amino Acid Residues in Polypeptides in a Single SERS Hot Spot. *Angewandte Chemie International Edition* **2020**, *59* (28), 11423–11431. https://doi.org/10.1002/anie.202000489.

(8) Zhao, Y.; Zhan, K.; Xin, P.-L.; Chen, Z.; Li, S.; De Angelis, F.; Huang, J.-A. Single-Molecule SERS Discrimination of Proline from Hydroxyproline Assisted by a Deep Learning Model. *Nano Lett* **2025**, *25* (18), 7499–7506. https://doi.org/10.1021/acs.nanolett.5c01177.

(9) Kirchberger-Tolstik, T.; Pradhan, P.; Vieth, M.; Grunert, P.; Popp, J.; Bocklitz, T. W.; Stallmach, A. Towards an Interpretable Classifier for Characterization of Endoscopic Mayo Scores in Ulcerative Colitis Using Raman Spectroscopy. *Anal Chem* **2020**, *92* (20), 13776–13784.
https://doi.org/10.1021/acs.analchem.0c02163.

(10) Chin, C.-L.; Chang, C.-E.; Chao, L. Interpretable Multiscale Convolutional Neural Network for
Classification and Feature Visualization of Weak Raman Spectra of Biomolecules at Cell Membranes. *ACS Sens* **2025**, *10* (4), 2652–2666. https://doi.org/10.1021/acssensors.4c03260.

jz-2025-017536.R2

Name: Peer Review Information for "Single-Molecule SERS Detection of Phosphorylation in Serine and Tyrosine Using Deep Learning-Assisted Plasmonic Nanopore"

Second Round of Reviewer Comments

Reviewer: 1

Comments to the Author

I have no further question.

Reviewer: 3

Comments to the Author

During the revision process, the authors carefully responded to each referee's comments, and the quality of the papers seemed to be improved. Therefore, I think the current paper could be acceptable for publication.

Author's Response to Peer Review Comments:

Dear Editor,

We have revised the manuscript to address the non-scientific changes for your re-consideration.

Best regards,
Jianan Huang, on behalf of all authors
--
Jian-An Huang, Ph.D.
Assistant Professor in Biosensors,
University of Oulu,
Room 2125B, Aapistie 5A, 90220 Oulu, Finland
Email: Jianan.huang@oulu.fi
Phone No.: +358 50 472 8456
